# Unilateral versus bilateral resistance training for explosive jump performance, linear sprint speed, and change-of-direction ability in male basketball players: a systematic review and meta-analysis

**DOI:** 10.3389/fphys.2026.1798477

**Published:** 2026-06-15

**Authors:** Ziyang Zhang, Fang Shu, Hongming Guo, Jingfeng Zhang, JianLei Gao

**Affiliations:** 1 Department of Physical Education, Foshan University, Foshan, Guangdong, China; 2 Sports Coaching College, Beijing Sports University, Beijing, China; 3 School of Education, Beijing Sport University, Beijing, China

**Keywords:** athletic performance, basketball players, explosive power, lower limb, unilateral training

## Abstract

**Background:**

While both unilateral (UT) and bilateral training (BT) are fundamental for enhancing athletic performance in basketball—a sport demanding high unilateral power and rapid changes of direction (COD)—equivocal evidence and a paucity of consensus exist regarding their comparative effectiveness on key sport-specific metrics.

**Objective:**

To resolve this ambiguity, we conducted a systematic review and meta-analysis to quantitatively compare the effects of UT versus BT on measures of explosive power, linear speed, and COD ability in basketball players.

**Methods:**

Adhering to PRISMA guidelines (PROSPERO: CRD420251149484), we systematically searched six databases until September 2025 for randomized controlled trials (RCTs) involving healthy basketball players. Nine RCTs (n=163 males) were included. Methodological quality was assessed using the Cochrane Risk of Bias tool. Data were synthesized using random-effects models in RevMan 5.4.1, calculating weighted mean differences (WMD) and 95% confidence intervals (CI). Heterogeneity (I^2^) and subgroup analyses were performed.

**Results:**

UT elicited superior improvements compared to BT in single-leg countermovement jump height (SL-CMJ: WMD = 0.64 cm, 95% CI [0.27, 1.00], p<0.001) and V-cut performance (WMD =–0.12 s, 95% CI [–0.23, –0.01], p=0.03). Conversely, no significant inter-group differences were found for bilateral CMJ, Reactive Strength Index (RSI), or linear sprint performance (5m and 20m). For the 505 agility test, high heterogeneity (I² = 95%) was observed, and no significant overall effect was found (WMD =–0.17 s, 95% CI: [–0.48,0.13]). For outcomes showing relatively high consistency, such as CMJ, additional subgroup and sensitivity analyses were performed based on age, intervention duration, and season phase. Among these analyses, subgroup analysis identified arm swing protocol inconsistency as a primary source of heterogeneity for CMJ outcomes.

**Conclusions:**

This meta-analysis demonstrates a clear task-specific superiority: UT is more effective for enhancing unilateral power and cutting agility, likely via neural and stability adaptations, whereas BT retains comparable efficacy for developing bilateral power and linear acceleration. These findings directly inform evidence-based, modality-specific strength and conditioning programming for basketball athletes.

**Systematic Review Registration:**

https://www.crd.york.ac.uk/PROSPERO/view/CRD420251149484, identifier CRD420251149484.

## 1 Introduction

Basketball is a high-intensity, intermittent sport characterized by frequent and explosive multi-directional movements, including sprinting, jumping, and rapid changes of direction (COD) (Ferioli et al., 2019; Zhang Z. et al., 2024). Success in these actions is fundamentally underpinned by the capacity for effective and rapid lower-limb force production, particularly in unilateral or asymmetrical stances during cutting, braking, and single-leg take-offs (Koyama et al., 2022; Pamuk et al., 2023). Consequently, optimizing lower-limb strength and power through targeted resistance training is a cornerstone of athletic preparation for basketball players (Zhang Z, et al., 2023). Two predominant modalities employed are unilateral training (UT), which stresses a single limb (e.g., split squats, single-leg hops), and bilateral training (BT), where both limbs work concurrently (e.g., back squats, bilateral jumps) (Liao et al., 2022).

A critical, yet unresolved question in strength and conditioning practice is determining the comparative effectiveness of these modalities for enhancing the distinct physical qualities required on the court. From a sport-specific biomechanical perspective, most high-impact offensive and defensive actions in basketball rely on unilateral loading, multi-planar stabilization, and independent neuromuscular control of single limbs, which cannot be fully replicated by bilateral, sagittal-plane dominant training (Struzik et al., 2014). From a neuromuscular perspective, unilateral tasks demand higher levels of intermuscular coordination, feedforward postural adjustments, and rate of force development under single-limb support, which are associated with specific neural adaptations including spinal reflex modulation, corticospinal excitability, and interhemispheric inhibition (Shin et al., 2009; Maestroni et al., 2026). Moreover, basketball-specific movements such as crossover cuts, single-leg landings, and step-back jumps require rapid switching between eccentric braking and concentric propulsion on one leg—a neuromuscular challenge that bilateral training may not adequately prepare the athlete for (Hewett et al., 2005). This combined biomechanical and neuromuscular rationale forms the core theoretical basis for comparing the transfer effects of UT and BT on basketball performance.

Both UT and BT offer distinct theoretical and practical advantages, supported by a growing body of research. BT permits the use of greater absolute loads, which is considered essential for maximizing maximal strength and bilateral power development—key contributors to vertical jump height and linear sprint acceleration (Villarreal et al., 2009; Zhang et al., 2025). Conversely, UT is posited to offer superior specificity to the unilateral nature of many sport-specific skills. The principle of training specificity suggests that UT may better enhance unilateral force application patterns, inter-limb coordination, and dynamic stability, potentially leading to greater improvements in single-leg power and COD ability (Belegišanin et al., 2025; Bobbert et al., 2006). Empirical studies have yielded mixed results. Some evidence indicates that UT may be more effective than BT for improving single-leg jump performance (Kidgell et al., 2011; Eliassen et al., 2018), sprinting (Young et al., 2002), and COD ability (Spiteri et al., 2014) in athletic populations. Notably, a recent study on youth basketball players reported unilateral flywheel training to be superior across strength, sprint, jump, and COD metrics (Andersen et al., 2014). However, this is contested by other findings showing no significant differences between UT and BT for bilateral jump performance, linear sprint speed, or certain agility tests (Morin et al., 2015; Cooley et al., 2024). These inconsistencies in the literature create a significant evidence gap, hindering the formulation of clear, evidence-based guidelines for coaches. The conflicting results are likely attributable to several moderating factors, including variations in participants’ training age, intervention protocols (modality, load, volume), and critically, a lack of standardization in outcome assessment methodologies (e.g., jump tests with or without arm swing) (Fitzpatrick et al., 2019; Chen et al., 2025).

Recent basketball-specific randomized controlled trials have reported inconsistent findings. For example, (Cao et al., 2024) found that unilateral plyometric training produced greater improvements in change-of-direction (COD) performance and reduced interlimb asymmetry compared with bilateral training in youth male basketball players. In contrast (Tao et al., 2025), reported greater improvements in vertical jump height after bilateral training, with no significant between-group difference in COD performance when training volume was matched. These discrepancies are unlikely to be explained solely by differences in training protocols, as the studies involved broadly similar participant ages, intervention durations, and outcome measures. The main uncertainty is whether unilateral training provides a specific advantage for COD and single-leg jump performance, while bilateral training may be more beneficial for bilateral vertical jump tasks, such as the countermovement jump, and sprint acceleration. Because neither modality has shown consistent superiority across basketball-specific outcomes, clearer evidence is still needed to guide training prescription.

Notably, existing reviews on this topic have been unable to resolve this uncertainty. The most comprehensive meta-analysis to date by Liao et al. (2022) made an important contribution demonstrating overall adaptations to unilateral versus bilateral resistance training across mixed athletic populations. However, because Liao et al. aggregated data from multiple sports without stratifying by type, their findings do not address whether the comparative effectiveness of UT and BT differs specifically for basketball players—a sport characterized by frequent unilateral take-offs, rapid lateral cuts, and defensive slides requiring single-limb stabilization under fatigue.

Thus, a basketball-specific synthesis restricted to male players does not merely replicate existing conclusions on a narrower population; rather, it provides a meaningful extension in three critical ways. First, it tests whether UT’s potential advantage for unilateral movements transfers to basketball-specific outcomes (e.g., single-leg jump, V-cut COD) that were diluted in mixed-sport meta-analyses. Second, it examines whether BT’s benefits for bilateral power remain significant in basketball players who frequently alternate between bilateral take-offs and unilateral landings. Third, it quantifies training effects specifically for male basketball players, whose training age and neuromuscular demands differ from female or mixed-sex samples. By answering whether UT or BT yields greater improvements in basketball-specific performance tests, this meta-analysis directly informs practitioners’ training decisions beyond the broader framework of Liao et al. (2022).

To date, no meta-analysis has exclusively examined basketball players to compare UT versus BT. Therefore, our study directly addresses this gap by exclusively synthesizing basketball-specific trial data, providing tailored, evidence-based recommendations for coaches. Given the heterogeneity among primary studies, narrative reviews or isolated randomized controlled trials (RCTs) are insufficient to provide definitive conclusions (Moran et al., 2021). A quantitative synthesis of the available evidence is urgently required. Systematic review with meta-analysis represents the highest level of evidence, capable of mathematically aggregating results from multiple RCTs to increase statistical power, estimate pooled effect sizes, and identify sources of heterogeneity (DeForest et al., 2014; Appleby et al., 2020). Recent meta-analyses in related areas have begun to clarify aspects of this debate but possess limitations. For instance, some have included broad athletic populations without sport-specific focus (Appleby et al., 2019), while others have not adequately accounted for methodological heterogeneity in test protocols (Flanagan and Comyns, 2008). A rigorously conducted, basketball-specific meta-analysis that systematically investigates and quantifies the differential effects of UT versus BT on a comprehensive set of performance outcomes—while accounting for key moderators like testing protocols—is therefore warranted to resolve existing contradictions and inform practice (Thapa et al., 2024; Li et al., 2025).

The primary objective of this systematic review and meta-analysis was to quantitatively synthesize the evidence from RCTs comparing the effects of UT and BT on measures of explosive power (bilateral and unilateral jumps, reactive strength), linear speed, and COD ability in basketball players. We aimed to determine: (1) whether UT or BT is superior for improving unilateral power (single-leg jump) and sport-specific COD tasks (e.g., V-cut); (2) whether differences exist in their effects on bilateral power and linear acceleration; and (3) to what extent methodological factors (e.g., jump test protocol) explain heterogeneity in the results. By adhering to PRISMA guidelines, employing rigorous study selection, and conducting subgroup analyses, this work seeks to provide a definitive, evidence-based answer to a key question in basketball strength and conditioning, ultimately guiding more precise and effective training prescriptions.

## 2 Methods

This meta-analysis was conducted in strict accordance with the Preferred Reporting Items for Systematic Reviews and Meta-Analyses (PRISMA) 2020 statement, and the protocol was prospectively registered with PROSPERO (Registration No. CRD420251149484). No deviations from the pre-registered protocol occurred during the study conduct (Gonzalo-Skok et al., 2019).

### 2.1 Search strategy

A comprehensive systematic search was performed across six electronic databases: PubMed, Web of Science, Scopus, Embase, the Cochrane Library, and EBSCO (Duan et al., 2024; Gonzalo-Skok et al., 2025). The search strategy utilized Boolean operators with the following key terms: (“unilateral training” OR “single-limb training” OR “unilateral resistance training”) AND (“bilateral training” OR “bilateral resistance training”) AND “basketball”. The search encompassed all records published up to September 2025. The full, database-specific Boolean search strings, aligned with the syntax rules of each database, are provided in **Supplementary Material 1** to ensure full transparency and reproducibility of the literature search.

### 2.2 Inclusion and exclusion criteria

Study eligibility was defined using the PICOS (Participants, Interventions, Comparisons, Outcomes, Study Design) framework (Cao et al., 2025; Shalom et al., 2023). Inclusion criteria were: (1) Participants were competitive basketball players who met all the following criteria: ① free from acute or chronic lower-limb injury in the 6 months prior to the study; ② had at least 1 year of systematic basketball-specific training experience, with a minimum training frequency of 2 sessions per week (≥90 minutes per session); ③ were registered athletes in formal competitive leagues (including youth competitive leagues, collegiate varsity teams, and professional youth teams), excluding recreational basketball participants. (2) the intervention consisted of a structured unilateral lower-limb resistance training program; (3) the comparator was a volume-matched bilateral lower-limb resistance training program (including standardized bilateral resistance protocols and routine bilateral lower-limb strength training programs with total training volume matched to the UT group); (4) reported outcomes included measures of lower-body power (e.g., jump height), sprint, or change-of-direction (COD) performance; and (5) the study design was a randomized controlled trial (RCT). Exclusion criteria comprised: (1) non-randomized or crossover study designs; (2) insufficient data for effect size calculation; (3) publication types such as reviews, meta-analyses, or conference abstracts; (4) unavailability of the full text; and (5) studies involving injured or non-athletic populations. Following screening, nine studies met all criteria for inclusion. (6) Recreational basketball participants without systematic competitive training experience.

### 2.3 Quality assessment

The methodological quality and risk of bias of the included studies were assessed independently by two reviewers using the revised Cochrane Risk of Bias tool for randomized trials (RoB 2). This tool evaluates bias across five domains: randomization process, deviations from intended interventions, missing outcome data, measurement of the outcome, and selection of the reported result. Judgments were categorized as ‘low risk’, ‘some concerns’, or ‘high risk’.

### 2.4 Statistical analysis

All statistical analyses were performed using Review Manager (RevMan) software, version 5.4.1 (Zemkova and Hamar, 2010). For continuous outcome data (reported as mean ± standard deviation), the weighted mean difference (WMD) with 95% confidence intervals (CIs) was calculated as the summary effect measure. Study heterogeneity was quantified using the I^2^ statistic, with an I^2^ value ≥ 50% indicating substantial heterogeneity and warranting the use of a random-effects model; otherwise, a fixed-effects model was applied (Wang et al., 2024). When substantial between-study heterogeneity was observed in the pooled analysis, pre-specified subgroup analyses were performed to explore potential sources of heterogeneity. For the CMJ outcome, subgroup analyses were mainly stratified according to clinically relevant study characteristics with sufficient available data, including participant age (adolescent: 13–19 years vs. young adult: 20–21 years), training status (competition period vs. offseason), and intervention duration (6 weeks vs. 8 weeks and above). Subgroup findings were interpreted primarily by comparing pooled effect estimates and heterogeneity patterns across strata. Methodological differences that were not suitable for formal subgroup analysis because of the limited number of studies, such as CMJ arm swing protocol, were addressed narratively in the Results and Discussion. The threshold for statistical significance was set at p < 0.05. For result interpretation, an effect was considered non-significant if the 95% CI crossed the line of no effect (zero). A 95% CI entirely above zero indicated a statistically significant positive effect favoring the unilateral training group, while a 95% CI entirely below zero indicated a significant effect favoring the bilateral training group. For all continuous outcomes with identical, directly comparable measurement units (centimeters for jump height, seconds for sprint/agility time) across included studies, the weighted mean difference (WMD) with 95% confidence intervals (CIs) was exclusively used as the summary effect measure. Standardized mean difference (SMD) was not applied in this analysis, as no outcomes required unit standardization.

### 2.5 Study selection and data extraction

Search results from all databases were imported into EndNote 21 reference management software for deduplication, which involved an automated process followed by manual verification (Zhou et al., 2024; Ramírez-Delacruz et al., 2022). Two investigators independently screened the titles, abstracts, and subsequently the full texts of potentially eligible studies against the pre-defined criteria. Any discrepancies were resolved through discussion or, if necessary, by consultation with a third reviewer to reach consensus. Data from the included studies were extracted using a standardized form. Data from the included studies were extracted using a standardized form, which covered participant baseline characteristics, complete unilateral and bilateral training protocol details (exercise type, intensity, volume, intervention duration, progression strategy, and training target), outcome measures, and key methodological parameters of the trials. Dual independent screening was performed for titles/abstracts and full texts.

### 2.6 Outcome measures

The primary outcomes of interest were measures of lower-body power and agility, specifically (Aztarain-Cardiel et al., 2025).

#### 2.6.1 505 agility test

Assesses change-of-direction speed and agility.

#### 2.6.2 Countermovement jump

Assesses bilateral lower-limb explosive power.

#### 2.6.3 Single-leg countermovement jump

Assesses unilateral explosive power and limb symmetry.

#### 2.6.4 Reactive strength index

Assesses the ability to transition rapidly from eccentric to concentric movement (e.g., in a drop jump).

#### 2.6.5 20-meter sprint

Assesses maximal linear sprint speed.

#### 2.6.6 5-meter sprint

Assesses short-distance acceleration.

#### 2.6.7 V-cut test

Assesses agility and rapid directional change in a specific movement pattern.

## 3 Results

### 3.1 Literature search

**Figure 1:**
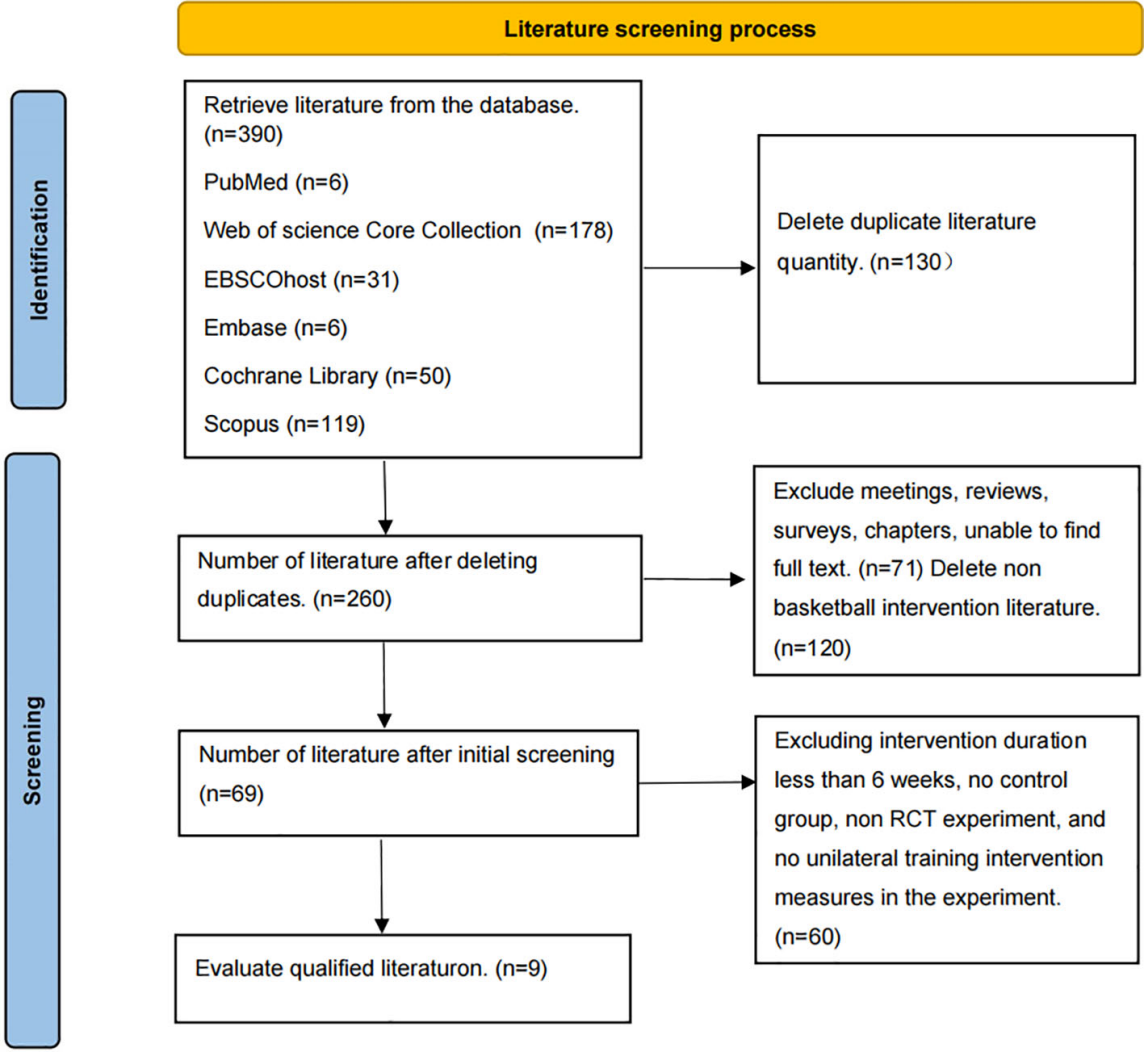
The preferred reporting items for systematic reviews and meta-analyses flow diagram.

The systematic literature search and screening process are summarized in **Figure 1**. The initial database searches identified 390 potentially relevant records. Following the removal of 130 duplicate entries, 260 unique records were screened based on their titles and abstracts. After applying the eligibility criteria at the full-text level, nine studies were deemed eligible for inclusion in the final meta-analysis.

### 3.2 Risk of bias assessment

**Figure 2:**
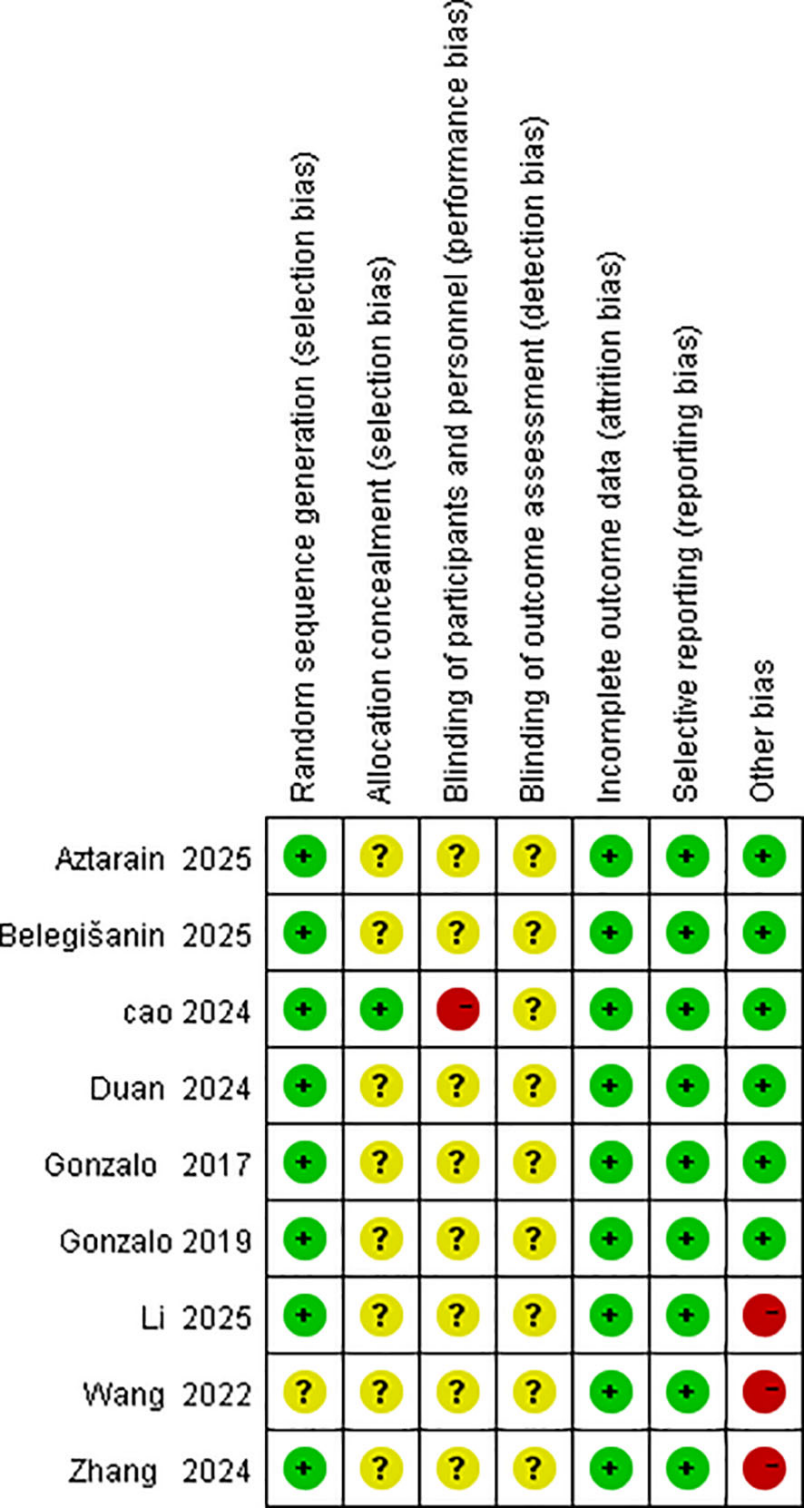
Risk of bias summary: a review of the authors’ judgments about the risk of bias of each item in each included study.

**Figure 3:**
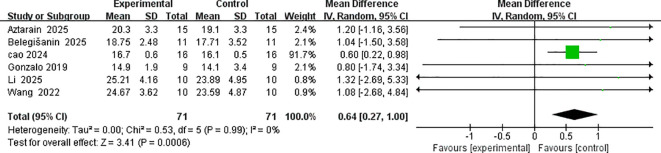
Forest plot of single-leg and double-leg performance in single-leg CMJ.

The methodological quality and risk of bias for the nine included studies were assessed. The summarized judgments across all bias domains are presented in **Figures 2** and **3**. Assessment indicated a predominantly low risk of bias concerning random sequence generation and the handling of missing outcome data. Conversely, an unclear risk of bias was frequently identified in key domains such as allocation concealment and the blinding of participants, personnel, and outcome assessors. While the overall methodological rigor of the included trials was acceptable, the lack of detailed reporting on concealment and blinding procedures introduces uncertainty and warrants cautious interpretation of the pooled results.

### 3.3 Characteristics of included studies

**Table 1** (BT control) and **Table 2** (UT experimental) summarize the included studies. In total, 163 male basketball players (age range 13–21 years) were pooled. All had at least one year of formal competitive experience. Five studies involved adolescents (13–19 years), and four focused on young adults (20–21 years). **Table 3** summarizes the unilateral (UT) and bilateral (BT) training interventions. All trials matched training volume, frequency, and duration between UT and BT groups (2–3 sessions/week, 6–10 weeks).

**Table 1:** Basic characteristics of the control group included in the study.

Study	Age(years)	Weight(kg)	Height(cm)	Sample size		Exercise	Frequency and week	Test used
						Content sets/repetitions		
Gonzalo-Skok et al., 2019	13.3 ± 06	59.1 ± 12.8	172.8 ± 7.9	9	9	Drop Jump 20 cm 3 × 5 SJ with arms swing 2 × 5 CMJ with arms swing 2 × 5 Tuck Jump 5 × 2 Hurdle jumps 3 × 5	2/6week	CMJ, 5 meter sprint, Single-leg CMJ, V-shaped directional change
Duan et al., 2024	20.80 ± 1.14	76.20 ± 8.70	183.00 ± 5.86	10	10	Barbell Back Squat 6x5 Bilateral Vertical Jump 6x10	2/8week	CMJ, 20 meter sprint, 505 Agility Test
Aztarain-Cardiel et al., 2025	14.2 (1.1)	182.3 (9.1)	68.3 (11.6)	15	15	Vertical acyclic (bilateral) Horizontal acyclic (bilateral) Vertical cyclic (bilateral) Horizontal cyclic (bilateral) Total volume (contacts/session)	2/6week	CMJ, Single-leg CMJ, V-shaped directional change, 20 meter sprint
Zhang W, et al., 2024	20.9 ± 0.9	69.1 ± 6.7	178.7 ± 5.8	15	15	Routine Training Plan for Both Lower Limbs	3/10week	CMJ
Cao et al., 2024	16.3 ± 0.8	68.9 ± 6.7	180.5 ± 5.0	16	16	Bilateral reactive pogo jumps (2x10); bilateral countermovement jumps (2x10); bilateral drop jumps at 10 cm (2x10) Bilateral horizontal jumps (2x10); bilateral three consecutive horizon jumps (2x10)	2/8week	Single-leg CMJ
Gonzalo-Skok et al., 2017	16.7 ± 1.7	74.9 ± 9.6	188.9 ± 7.5	9	9	Bilateral 90° Deep Squat 2x5 Bilateral Vertical Jump 2x5 Jump in place with both sides 2x5	2/6week	CMJ, 5 meter sprint, V-shaped directional change
Wang 2022	20.4 ± 1.35	80.71 ± 5.02	186.1 ± 4.06	10	10	Vertical, horizontal, and forward leg jumps 2x4	2/8week	CMJ, Single-leg CMJ, V-shaped directional change, RSI
Belegišanin et al., 2025	15.2 ± 0.4	69 ± 7	183 ± 4	11	11	Half Squat (Flywheel Device) 6x6	2/6week	CMJ, Single-leg CMJ, RSI, 20 meter sprint, 5 meter sprint, 505 Agility Test
Li et al., 2025	20.4 ± 1.4	80.7 ± 5.0	186.1 ± 4.1	10	10	B vertical jump and freezing 2x8 B standing long jump and freezing 2x8 B consecutive vertical jump 2x8	2/8week	CMJ, Single-leg CMJ, V-shaped directional change, RSI, 505 Agility Test

**Table 2:** Basic characteristics of the experimental group included in the study.

Study	Age(years)	Weight(kg)	Height(cm)	Sample size		Exercise	Frequency and week	Test used
						Content sets/repetitions		
Gonzalo-Skok et al., 2019	13.3 ± 06	59.6 ± 11.7	171.7 ± 7.2cm	9	9	Drop Jump 10 cm3 × 5 SLJ2 × 5 SLJ without CMJ2 × 5 Unilateral jumps 5 × 2 Triple jumps 3 × 5	2/6week	CMJ, 5 meter sprint, Single-leg CMJ, V-shaped directional change
Duan et al., 2024	19.90 ± 1.45	77.90 ± 5.90	183.20 ± 6.82	10	10	Bulgarian Split Squat 5x6 Reverse Lunge Jump 10x5	2/8week	CMJ, 20 meter sprint, 505 Agility Test
Zhang W, et al., 2024	20.9 ± 1.1	73.5 ± 5.3	182.1 ± 4.1	15	15	Bulgarian split squat 3x6 Box step-up 3x12 Single-leg calf raise 1x12 Plyometric jump 1x12 Single-leg jump with rear leg lift 1x12 Single-leg lateral jump 1x12 Single-leg consecutive jumps 1x12	3/10week	CMJ
Gonzalo-Skok et al., 2017	16. 8 ± 1.7	76.9 ± 8.6	190.4 ± 6.9	9	9	Single-Leg 90° Deep Squat 3x5 Single-leg vertical jump 2x5 Single-Side Jump from the Spot 2x5	2/6week	CMJ, 5 meter sprint, V-shaped directional change
Aztarain-Cardiel et al., 2025	14.1 (1.3)	69.1 ± 9.3	181.1 ± 8.0	15	15	Vertical acyclic Horizontal acyclic Vertical cyclic (unilateral) Horizontal cyclic (unilateral)	2/6week	CMJ, Single-leg CMJ, V-shaped directional change, 20 meter sprint
Belegišanin et al., 2025	15.5 ± 0. 5	71 ± 12	186 ± 6	11	11	Split Squat (Flywheel Device)6x6	2/6week	CMJ, Single-leg CMJ, RSI, 20 meter sprint, 5 meter sprint, 505 Agility Test
	15.9 ± 0.9	62.4 ± 5.2	175.5 ± 6.9	16	16	Single-Sided Lateral Jump 2x5 Single-Sided Triple Jump 2x5 Single-leg reactive jump 2x5 Single-Leg Squat Jump 2x10 Single-leg landing jump 2x10	2/8week	Single-leg CMJ
Zhiguang, 2022	20.6 ± 1.51	79.95 ± 6.56	185.6 ± 3.63	10	10	Single-leg jumps: vertical, horizontal, and forward	2/8week	CMJ, Single-leg CMJ, V-shaped directional change, RSI
Li et al., 2025	20.6 ± 1.5	79.9 ± 6.6	185.6 ± 3.6	10	10	U vertical jump and freezing 2x8 U standing long jump and freezing 2x8 U consecutive vertical jump 2x8	2/8week	CMJ, Single-leg CMJ, V-shaped directional change, RSI, 505 Agility Test

**Table 3:** Summary of unilateral (UT) and bilateral (BT) training interventions across included studies.

Study	Group	Core strength exercises	Core plyometric exercises	Training intensity	Sets × repetitions	Frequency & total duration	Training target
Gonzalo-Skok et al., 2019	BT	——	Bilateral drop jump (20cm), bilateral CMJ with arm swing, tuck jump, bilateral hurdle jumps	Bodyweight	2–5 sets × 2–10 reps	2 sessions/week, 6 weeks	Power
Gonzalo-Skok et al., 2019	UT	——	Unilateral drop jump (10cm), single-leg jump, unilateral triple jump	Bodyweight	2–5 sets × 2–10 reps	2 sessions/week, 6 weeks	Power
Duan et al., 2024	BT	Barbell back squat	Bilateral vertical jump	60–80% 1RM	5–6 sets × 5–10 reps	2 sessions/week, 8 weeks	Mixed Strength-Power
Duan et al., 2024	UT	Bulgarian split squat	Reverse lunge jump	60–80% 1RM	5–10 sets × 5–6 reps	2 sessions/week, 8 weeks	Mixed Strength-Power
Aztarain-Cardiel et al., 2025	BT	——	Bilateral vertical/horizontal acyclic & cyclic jumps	Bodyweight	Matched to UT group	2 sessions/week, 6 weeks	Power
Aztarain-Cardiel et al., 2025	UT	——	Unilateral vertical/horizontal acyclic & cyclic jumps	Bodyweight	Matched to BT group	2 sessions/week, 6 weeks	Power
Zhang W, et al., 2024	BT	Routine bilateral lower-limb strength training	——	60–75% 1RM	Matched to UT group	3 sessions/week, 10 weeks	Strength
Zhang W, et al., 2024	UT	Bulgarian split squat, box step-up, single-leg calf raise	Single-leg plyometric jumps, single-leg lateral jumps	60–75% 1RM	1–3 sets × 6–12 reps	3 sessions/week, 10 weeks	Mixed Strength-Power
	BT	——	Bilateral reactive pogo jumps, bilateral CMJ, bilateral drop jump (10cm), bilateral horizontal jumps	Bodyweight	2 sets × 10 reps	2 sessions/week, 8 weeks	Power
	UT	——	Single-leg lateral jump, single-leg triple jump, single-leg reactive jump, single-leg squat jump	Bodyweight	2 sets × 5–10 reps	2 sessions/week, 8 weeks	Power
Gonzalo-Skok et al., 2017	BT	Bilateral 90° deep squat	Bilateral vertical jump, bilateral in-place jump	60–70% 1RM	2 sets × 5 reps	2 sessions/week, 6 weeks	Mixed Strength-Power
Gonzalo-Skok et al., 2017	UT	Single-leg 90° deep squat	Single-leg vertical jump, unilateral in-place jump	60–70% 1RM	2–3 sets × 5 reps	2 sessions/week, 6 weeks	Mixed Strength-Power
Zhiguang, 2022	BT	——	Bilateral vertical/horizontal/forward jumps	Bodyweight	2 sets × 4 reps	2 sessions/week, 8 weeks	Power
Zhiguang, 2022	UT	——	Single-leg vertical/horizontal/forward jumps	Bodyweight	Matched to BT group	2 sessions/week, 8 weeks	Power
Belegišanin et al., 2025	BT	Bilateral half squat (flywheel device)	——	Flywheel resistance, matched to UT	6 sets × 6 reps	2 sessions/week, 6 weeks	Strength
Belegišanin et al., 2025	UT	Split squat (flywheel device)	——	Flywheel resistance, matched to BT	6 sets × 6 reps	2 sessions/week, 6 weeks	Strength
Li et al., 2025	BT	——	Bilateral vertical jump with isometric hold, bilateral standing long jump with isometric hold, bilateral consecutive vertical jump	Bodyweight	2 sets × 8 reps	2 sessions/week, 8 weeks	Power
Li et al., 2025	UT	——	Unilateral vertical jump with isometric hold, unilateral standing long jump with isometric hold, unilateral consecutive vertical jump	Bodyweight	2 sets × 8 reps	2 sessions/week, 8 weeks	Power

#### 3.3.1 UT protocols

Unilateral exercises fell into two categories: strength−oriented (Bulgarian split squats, reverse lunges, single−leg step−ups, single−leg half squats on a flywheel, single−leg calf raises) and plyometric/power−oriented (single−leg countermovement jumps, drop jumps, horizontal/vertical/lateral hops, unilateral triple jumps). Seven of nine studies used a mixed strength−power design; the other two focused solely on unilateral plyometric power. Intensity was prescribed via relative load (60–85% 1RM) for strength work, and bodyweight with fixed drop heights or set−rep schemes for plyometrics. Progressive overload was achieved by adding weight, repetitions, or movement difficulty.

#### 3.3.2 BT protocols

Bilateral exercises similarly included strength moves (barbell back squats, bilateral 90° deep squats, bilateral half squats on a flywheel) and plyometric moves (bilateral countermovement jumps, drop jumps, tuck jumps, horizontal jumps, hurdle jumps). Intensity, progressive overload strategies, and set−rep volumes were matched to the UT group. All BT interventions used a mixed strength−power design comparable to their paired UT group.

Interventions were delivered 2–3 times per week for 6–10 weeks. Experimental groups performed unilateral lower−limb training; controls performed bilateral training. One study (Zhang W, et al., 2024) used a routine volume−matched bilateral program as control rather than an isolated BT protocol, but it was retained because training frequency, volume, and duration were fully matched.

### 3.4 Participants and outcome measures

Five studies recruited adolescents (13–19 years) and four young adults (20–21 years). Outcome measures included: countermovement jump (CMJ, n=7), single−leg CMJ (n=6), V−cut test (n=5), reactive strength index (RSI, n=3), 505 agility test (n=3), 20−m sprint (n=3), and 5−m sprint (n=3). All studies confirmed participants were healthy and injury−free during the intervention.

### 3.5 Single-leg CMJ

Six studies examined the effects of unilateral versus bilateral training on SL-CMJ performance. Heterogeneity among these studies was negligible ( I²=0%;χ²=0.53,df=5,p=0.99), warranting the use of a fixed-effects model for meta-analysis. The pooled analysis revealed a statistically significant overall effect in favor of unilateral training( Z=3.41,p=0.0006). The weighted mean difference (WMD) was 0.64 cm (95%CI: [0.27,1.00]), indicating that unilateral training produced a greater improvement in single-leg jump height compared to bilateral training. This 0.64 cm improvement in SL-CMJ height has meaningful on-court relevance for basketball players: prior basketball-specific research has shown that even 0.5 cm gains in single-leg jump height are associated with higher layup finishing success rates and improved contested rebound acquisition (Theodorou et al., 2022), as marginal vertical take-off gains directly reduce shot blocking risk and expand access to rebounding opportunities. This effect size is also consistent with previously reported meaningful, practically relevant changes in single-leg jump performance following 6–10 weeks of targeted training in adolescent and young adult basketball players (See **Figure 3** for details).

### 3.6 V-shaped directional change

**Figure 4:**
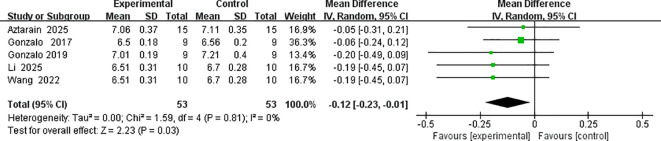
Forest plot of single-leg and double-leg performance in V-shaped directional change.

Five studies assessed V-cut performance. No significant heterogeneity was observed( I²=0%,χ²=1.59,df=4,p=0.81), and a fixed-effects model was applied. The overall effect was statistically significant( Z=2.23,p=0.03). The WMD was -0.12s,  95%CI: [−0.23,−0.01], demonstrating that unilateral training led to a significantly greater reduction in completion time (i.e., improved change-of-direction speed) relative to bilateral training (See **Figure 4** for details).

### 3.7 RSI

**Figure 5:**
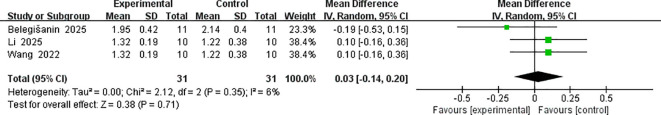
Forest plot of single-leg and double-leg performance in RSI.

Three studies compared the effects of training modalities on the Reactive Strength Index. Heterogeneity was low( I²=6%;χ²=2.12,df=2,p=0.35); thus, a fixed-effects model was applied. The analysis found no statistically significant difference between groups, with a weighted mean difference (WMD of 0.03 95%CI: [−0.14,0.20]). As the confidence interval crosses zero, the result suggests that neither unilateral nor bilateral training is superior for improving RSI (See **Figure 5** for details).

### 3.8 505 agility test

**Figure 6:**
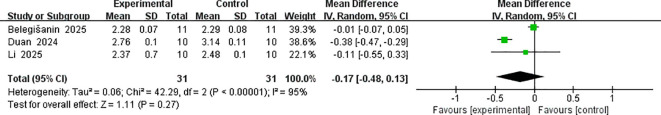
Forest plot of single-leg and double-leg performance in 505 Agility.

Three studies assessed 505 Agility Test performance. Substantial between-study heterogeneity was observed (I² = 95%; χ² = 42.29, df = 2, p < 0.00001), suggesting considerable variability across studies. Although a random-effects model was used, the pooled estimate (WMD = –0.17 s; 95% CI:[–0.48,0.13]) should be interpreted cautiously given the high heterogeneity and the limited evidence currently available for this outcome. Findings at the individual study level were not entirely consistent: two studies reported relatively small between-group differences, whereas one study (Duan et al., 2024) showed a comparatively larger effect in favor of UT. Leave-one-out sensitivity analysis suggested that this study may have been a major contributor to the observed heterogeneity. In addition, although all included studies were classified as using a “505” agility test, the specific test protocols were not fully standardized. Differences in timing-gate placement, approach distance before the 180° turn, definition of the timed interval, and outcome derivation methods may have influenced the relative contribution of sprinting, braking, turning, and re-acceleration components, which may partly account for the substantial heterogeneity observed for this outcome (See **Figure 6** for details).

### 3.9 20 meter sprint

**Figure 7:**
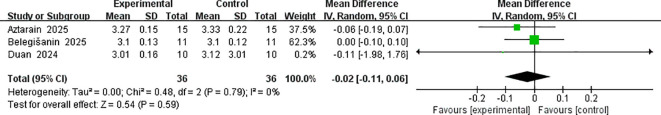
Forest plot of single-leg and double-leg performance in 20 meter sprint.

Three studies assessed 20-meter sprint performance. No heterogeneity was detected( I²=0%;χ²=0.48,df=2,p=0.79), and a fixed-effects model was employed. The meta-analysis showed no significant difference between training modalities (WMD = -0.02 s; 95% CI:[–0.11, 0.06]). The confidence interval spanning zero indicates that neither unilateral nor bilateral training was superior for improving 20-meter sprint time (See **Figure 7** for details).

### 3.10 5 meter sprint

**Figure 8:**
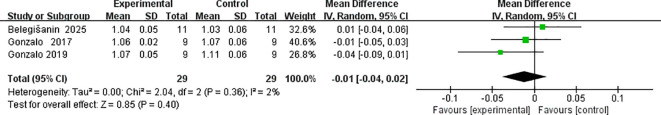
Forest plot of single-leg and double-leg performance in 5 meter sprint.

Three studies evaluated 5-meter sprint performance. Heterogeneity was negligible( I²=2%;χ²=2.04,df=2,p=0.36), and a fixed-effects model was used. No statistically significant effect was found (WMD = -0.01 s; 95% CI:[–0.04,0.02]). This result suggests that both unilateral and bilateral training elicit comparable effects on short-distance acceleration (See **Figure 8** for details).

### 3.11 CMJ

**Figure 9:**
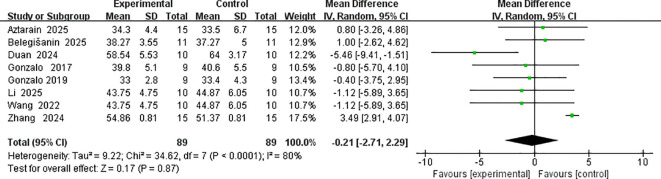
Forest plot of single-leg and double-leg performance in CMJ.

Eight studies evaluated bilateral CMJ performance. Substantial heterogeneity was present among the studies ( I²=80%;χ²=34.62,df=7,p<0.0001). Using a random-effects model, the meta-analysis found no statistically significant difference between training groups ( WMD=−0.21cm;95% CI:[−2.71,2.29]. The confidence interval spanning zero indicates that unilateral training did not elicit superior improvements in bilateral jump height compared to bilateral training (See **Figure 9** for details).

### 3.12 Subgroup analysis

**Figure 10:**
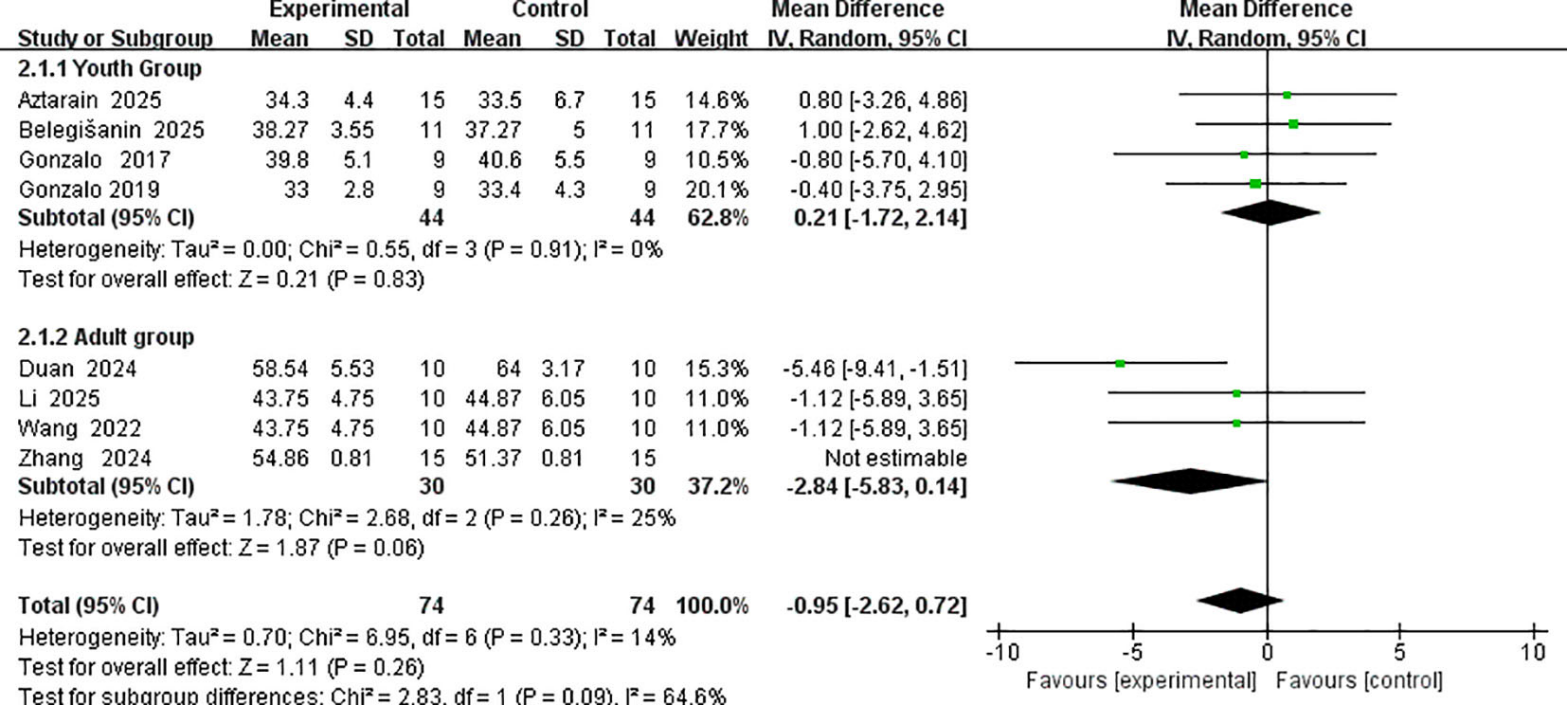
Subgroup analysis in CMJ test (age group).

Given the substantial heterogeneity in the pooled bilateral CMJ outcome (I²= 80%), we conducted subgroup analyses to explore potential sources of variability. In the age-based analysis, the pooled effect was WMD = 0.21 cm,95% CI: [–1.72,2.14] with low heterogeneity (I²= 0%) in the youth subgroup (4 studies), and WMD = –2.84 cm (95% CI:[–5.83, 0.14]) with(I²= 25%) in the adult subgroup (4 studies). Although the adult subgroup showed a relatively larger effect estimate, the between-subgroup difference was not statistically significant, suggesting that age may partly explain between-study variation but does not support a clear age-related subgroup effect (See **Figure 10** for details).

**Figure 11:**
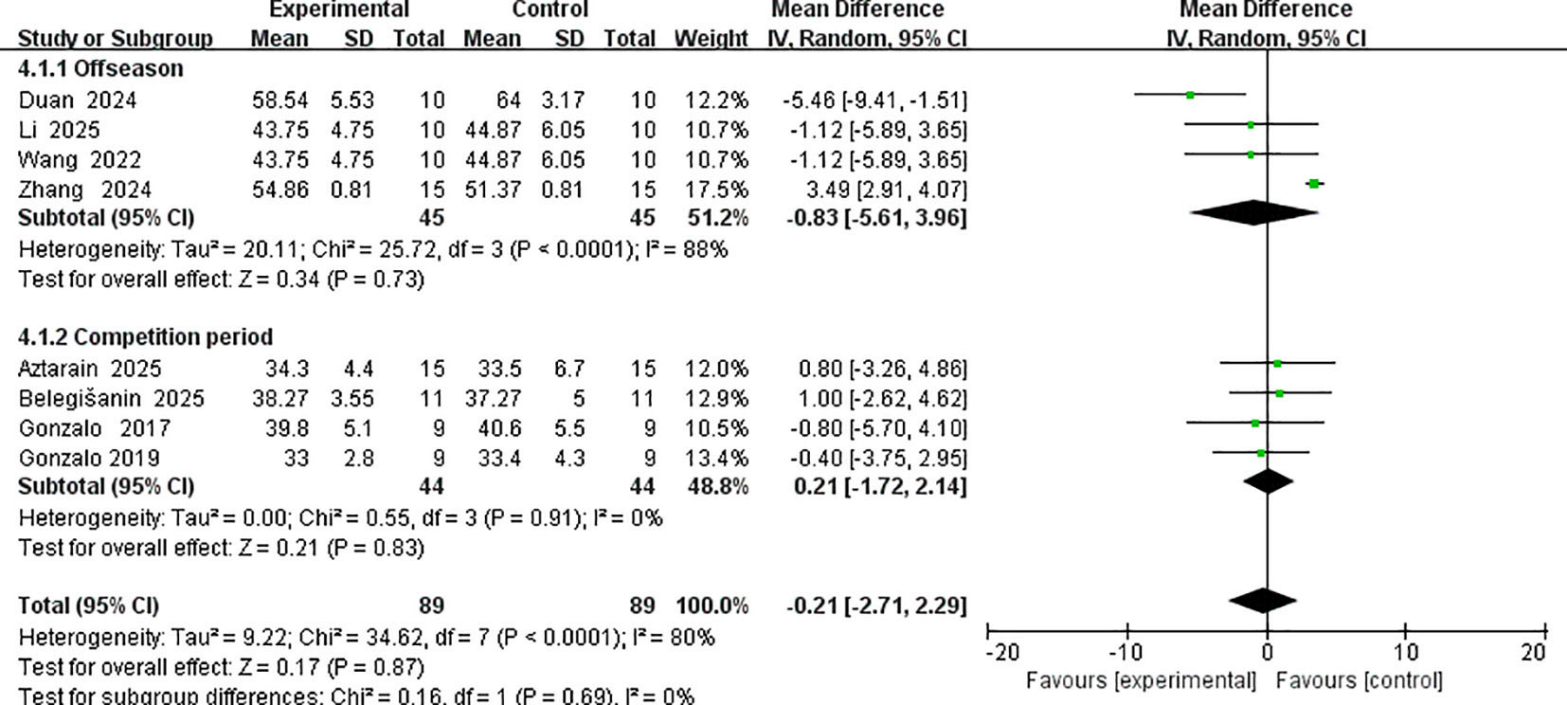
Subgroup analysis in CMJ test (training status group).

In the subgroup analysis by training status, the pooled effect in the offseason subgroup was WMD = –0.83 cm (95% CI: [–5.61,3.96]), with substantial heterogeneity (I²= 88%), whereas the pooled effect in the competition-period subgroup was WMD = 0.21 cm (95% CI:[–1.72,2.14]), with low heterogeneity (I²= 0%). This suggests greater variability among offseason studies, which may be related to differences in training load distribution, baseline physical condition, and phase-specific training objectives. In contrast, training during the competition period is usually more stable, which may partly explain the greater consistency of results. However, no stable between-subgroup difference was observed (See **Figure 11** for details).

Reasons for downgrading: (1) allocation concealment and/or blinding was unclear in most included studies; (2) the pooled effect estimate had a wide 95% CI crossing the line of no effect; and (3) heterogeneity was relatively high.

**Figure 12:**
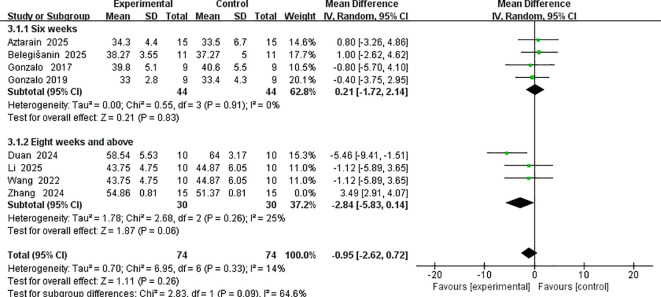
Subgroup analysis in CMJ test (training duration group).

In the subgroup analysis by intervention duration, the pooled effect was WMD = 0.21 cm, (95% CI: [–1.72,2.14]; I²= 0%) in the 6-week subgroup and WMD = –2.84 cm (95% CI: [–5.83, 0.14]; I²= 25%) in the ≥8-week subgroup. Further examination showed that (Zhang W, et al., 2024) differed from the other studies in the ≥8-week subgroup in terms of training dose, as its intervention lasted 10 weeks and was delivered three times per week, whereas the other studies more commonly used 8-week protocols with two sessions per week. After excluding (Zhang W, et al., 2024), heterogeneity decreased markedly, suggesting that differences in training dose may have contributed to the between-study heterogeneity. However, given the limited number of included studies and the lack of complete consistency in intervention design across studies, these findings should be interpreted with caution and considered exploratory (See **Figure 12** for details).

We also re-examined CMJ testing protocols across studies. Most studies used a standardized hands-on-hips procedure, which helps reduce upper-body momentum and improve comparability; previous studies and reviews have likewise emphasized the importance of standardized testing when interpreting jumping performance in basketball players (Shalom et al., 2023; Zhou et al., 2024). In contrast (Duan et al., 2024), allowed a free arm swing, which may better reflect basketball-specific jumping actions but may also enhance jump performance through upper-body contribution, thereby affecting direct comparability.

However, because only one study used the free arm swing protocol, we did not perform a formal subgroup analysis on this basis. Moreover, excluding Duan et al. (2024) did not materially reduce heterogeneity, suggesting that arm swing protocol alone is unlikely to fully explain the observed heterogeneity. Therefore, the high heterogeneity in CMJ outcomes is more likely due to the combined influence of participant age, training background, intervention duration, and testing procedures rather than a single factor. Overall, although testing standardization may have influenced effect estimates, the available evidence still does not indicate a consistent or clear difference between unilateral and bilateral training in improving bilateral CMJ performance.

### 3.13 Certainty of evidence (GRADE)

GRADE Working Group grades of evidence:

High quality: Further research is very unlikely to change our confidence in the estimate of effect. Moderate quality: Further research is likely to have an important impact on our confidence in the estimate of effect and may change the estimate. Low quality: Further research is very likely to have an important impact on our confidence in the estimate of effect and is likely to change the estimate. Very low quality: We are very uncertain about the estimate.

The certainty of evidence was assessed using the GRADE approach (**Table 4**). The evidence was rated as moderate for single-leg CMJ and V-shaped change-of-direction performance, low for RSI and sprint outcomes over 5 m and 20 m, and very low for the 505 agility test and CMJ. Downgrading was mainly due to methodological limitations in the included studies, particularly unclear allocation concealment and blinding. For some outcomes, imprecision, as indicated by wide confidence intervals, and inconsistency due to relatively high heterogeneity also contributed to lower certainty ratings.

**Table 4:** GRADE evidence summary.

Outcome	Studies (k)	Participants (n)	Effect estimate (WMD, 95% CI)	Certainty of evidence (GRADE)	Comments/reasons for downgrading
Single-leg CMJ	6	142	WMD = 0.64 (95% CI 0.27 to 1.00)	⊕⊕⊕◯ MODERATE (1)	due to risk of bias
V-shaped directional change	5	106	WMD = -0.12 (95% CI -0.23 to -0.01)	⊕⊕⊕◯ MODERATE (1)	due to risk of bias
RSI	3	62	WMD = 0.03 (95% CI -0.14 to 0.20)	⊕⊕◯◯ LOW (1,2)	due to risk of bias, imprecision
505 Agility Test	3	62	WMD = -0.17 (95% CI -0.48 to 0.13)	⊕◯◯◯ VERY LOW (1,2,3)	due to risk of bias, inconsistency, imprecision
20 meter sprint	3	72	WMD = -0.02 (95% CI -0.11 to 0.06)	⊕⊕◯◯ LOW (1,2)	due to risk of bias, imprecision
5 meter sprint	3	58	WMD = -0.01 (95% CI -0.04 to 0.02)	⊕⊕◯◯ LOW (1,2)	due to risk of bias, imprecision
CMJ	8	178	WMD = -0.21 (95% CI -2.71 to 2.29)	⊕◯◯◯ VERY LOW (1,2,3)	due to risk of bias, inconsistency, imprecision

## 4 Discussion

### 4.1 Main findings and training specificity

This systematic review and meta-analysis compared the effects of unilateral training (UT) and bilateral training (BT) in basketball players. Nine randomized controlled trials involving 163 male players were included. Outcomes covered unilateral and bilateral explosive power, linear sprint speed, and change-of-direction (COD) ability. The results showed a task-specific pattern. Compared with BT, UT led to greater improvements in single-leg countermovement jump height (WMD = 0.64 cm, 95% CI [0.27, 1.00], p = 0.0006) and V-cut performance (WMD = −0.12 s, 95% CI [−0.23, −0.01], p = 0.03). No significant between-group differences were found for bilateral CMJ, reactive strength index (RSI), or 5-m and 20-m sprint performance. For the 505 agility test, a reliable pooled conclusion could not be made because heterogeneity was high (I² = 95%) and few studies were available. These findings are consistent with the principle of training specificity, suggesting that transfer may depend on the biomechanical and neuromuscular similarity between the exercise and the performance task.

### 4.2 Mechanistic interpretation of key results

#### 4.2.1 Superior effects of unilateral training

The greater effects of unilateral training (UT) on SL-CMJ and V-cut performance are consistent with the biomechanical and neuromuscular demands of basketball. The V-cut is a common off-ball action used to create space. During its braking phase, unilateral hip abductors and knee stabilizers show 2–3 times greater activation than in bilateral squats, helping to control medial knee displacement and trunk rotation (Jòdar-Portas et al., 2023). UT may therefore provide a more specific stimulus to these stabilizing muscles and to feedforward neuromuscular control, improving the eccentric–concentric transition required for change-of-direction performance (Shi et al., 2022).

The advantage of UT in single-leg jump performance also reflects the movement pattern of basketball competition. More than 70% of jump take-offs in games, including layups, driving jumps, and contested rebounds, are performed unilaterally and depend on single-limb neural drive rather than bilateral force production (Cao et al., 2024). This may be partly related to the bilateral deficit, whereby simultaneous force production by both limbs is lower than the summed force produced by each limb separately (Bobbert et al., 2006). Because UT requires each limb to generate force independently under relatively high load, it may enhance neural drive, motor unit recruitment, and inter-muscular coordination specific to unilateral force production (Zhao et al., 2024). Neurophysiological evidence also suggests that UT can increase corticospinal excitability in both hemispheres and facilitate neural drive to the trained limb (Kidgell et al., 2011). This is supported by electromyographic findings showing greater activation of prime movers, such as the quadriceps, during unilateral squats than during bilateral variations (Eliassen et al., 2018).

#### 4.2.2 Comparative effects of bilateral training

The lack of significant between-group differences in bilateral CMJ, RSI, and linear sprint performance should be interpreted with caution. These findings should not be taken as evidence that UT and BT are fully equivalent; rather, they indicate that the available evidence did not show a clear advantage of either modality for these outcomes.

Bilateral exercises, such as back squats, allow greater absolute loads and are therefore an important stimulus for maximal strength and rate of force development, both of which contribute to vertical jumping and linear acceleration (Villarreal et al., 2009; Zhang et al., 2025). The absence of a significant difference between UT and BT in bilateral CMJ improvement is consistent with Zhang et al. (2023), suggesting that both approaches may be sufficient to enhance bilateral force production. Similarly, the comparable effects on sprint performance may relate to force-vector specificity (Fitzpatrick et al., 2019). Although sprinting involves alternating single-leg actions, its main mechanical demand is the production of horizontal ground reaction force. Bilateral exercises with a horizontal force vector, such as sled pushes, can effectively develop this capacity (Chen et al., 2025). When training intensity and volume are comparable, both UT and BT may improve the strength-power qualities underlying sprint performance (Moran et al., 2021).

For the 505 agility test, the inconsistent findings across studies may be partly due to differences in testing procedures, including timing gate placement, sprint distance before the turn, and scoring methods. Future studies using standardized protocols are needed to clarify whether UT and BT differ in their effects on this outcome.

### 4.3 Heterogeneity sources and moderator variable analysis

To examine potential sources of between-study heterogeneity, we performed pre-specified moderator analyses for eligible outcomes, including participant age, competitive level, intervention duration, and sex. Training frequency was not examined as a moderator because it showed little variation across protocols.

For bilateral CMJ, substantial heterogeneity was observed (I² = 80%). Subgroup analyses were conducted by age, training status, and intervention duration. The adult subgroup showed a larger effect estimate than the youth subgroup, but the between-subgroup difference was not significant, indicating no clear age-related moderation. Greater variability was also seen in offseason and longer-duration interventions, whereas competition-period and 6-week studies showed lower heterogeneity. However, these patterns were not accompanied by consistent between-subgroup differences, and the overlap between training status and intervention duration limited further interpretation. Differences in CMJ testing procedures, such as the use of arm swing, may also have contributed. Overall, heterogeneity in bilateral CMJ outcomes likely reflects the combined influence of participant characteristics, intervention design, and testing methods rather than a single moderator.

For the 505 agility test, heterogeneity was very high (I² = 95%), and the pooled estimate should be interpreted cautiously. As noted above, studies labelled as “505” tests differed in protocol details, including timing-gate placement, approach distance, recorded interval, and scoring method. These differences may have changed the relative contribution of sprinting, braking, turning, and re-acceleration, thereby contributing to the observed variability.

Sex could not be assessed as a moderator because all included trials involved male basketball players. Taken together, the findings suggest that the main conclusions are generally consistent across common variations in participant and training characteristics within the available evidence on male basketball players.

### 4.4 Alignment with existing literature and theoretical contributions

The results of this meta-analysis are consistent with previous work on unilateral and bilateral training and add basketball-specific evidence. The greater effect of UT on unilateral power, as reflected by SL-CMJ, supports the view that unilateral exercises provide more specific neural and mechanical stimuli for single-leg tasks. This finding is also in line with Liao et al. (2022), who reported larger effects of UT on single-leg jump performance in general athletic populations. The present study extends that evidence by showing a similar pattern in basketball players, whose performance depends heavily on single-leg jumping, cutting, and rapid changes of direction.

The findings also support key principles in strength and conditioning. According to the specificity principle, training adaptations are greater when exercises resemble the target movement. UT better reflects the unilateral loading and multi-planar stability demands of basketball actions such as cutting and single-leg jumping, which may explain its advantage in unilateral performance. In contrast, BT is more consistent with bilateral, sagittal-plane force production and therefore produced comparable improvements in outcomes such as bilateral jumping and linear sprinting. These results are also compatible with the force-vector theory (Fitzpatrick et al., 2019), which suggests that transfer is influenced by the direction of force application. UT places greater demands on frontal- and transverse-plane control, which is relevant to basketball-specific change-of-direction tasks, whereas many BT exercises mainly emphasize vertical or sagittal-plane force production.

Finally, these findings contribute to the discussion on bilateral deficit and inter-limb asymmetry. The advantage of UT for single-leg performance suggests that it may be useful for improving unilateral capacity and addressing side-to-side strength differences, which have been associated with injury risk and reduced performance. Because each limb works independently during UT, adaptations in the weaker limb are less likely to be masked by compensation from the stronger limb, a limitation that may occur in some bilateral exercises.

### 4.5 Practical applications for basketball strength and conditioning

These findings support the combined use of UT and BT in male basketball players, with training selection based on the main goal of each phase rather than treating the two methods as mutually exclusive.

In annual periodized programs, BT may be emphasized during the general preparatory phase, when the aim is to develop maximal strength and bilateral power. At this stage, BT can account for approximately 60–70% of lower-body training volume, including exercises such as back squats and bilateral plyometrics, while UT can account for 30–40% to address inter-limb asymmetry and develop basic unilateral stability. During the specific preparatory and pre-competition phases, the focus should shift toward sport-specific transfer. In these phases, UT may account for approximately 60–70% of lower-body training volume to improve unilateral power and V-cut agility, whereas BT can be maintained at 30–40% to preserve general strength and linear sprint capacity.

UT may be particularly useful for players with unilateral strength deficits or COD limitations, adolescent athletes developing dynamic stability and inter-limb coordination, and in-season athletes who require sport-specific stimulus with relatively lower systemic fatigue. Overall, BT provides the force foundation for jumping and sprinting, while UT helps transfer this capacity to basketball-specific single-leg jumps and multi-planar cutting actions.

### 4.6 Limitations

Several limitations must be acknowledged. The total sample (163 participants across nine studies) is modest, which limited statistical power for outcomes with fewer studies and precluded multi−factor meta−regression or dose−response analysis. Extreme heterogeneity for the 505 test reflects a lack of standardized agility testing across primary studies. A major limitation is the exclusive inclusion of male players; no female basketball players were represented. Female athletes exhibit different neuromuscular adaptations, injury risk profiles, and training responsiveness (MoTrPAC Study Group et al., 2024; Hassan, 2025; Bullock et al., 2025; Stojanović et al., 2025), so our findings cannot be reliably generalized to women. Age−related analyses were also constrained – only five studies in adolescents (13−19 years) and four in young adults (20−21 years), too few for robust subgroup meta−regression (McQuilliam et al., 2020; Calle et al., 2025). Finally, we only examined physical performance outcomes; long−term effects on injury prevention, retention of adaptations, or actual in−game statistics (rebounds, steals, scoring efficiency) remain unaddressed.

### 4.7 Future directions

Future research should prioritize large−scale RCTs with detailed training logs, standardized agility testing, studies in female p layers and other underrepresented groups, longitudinal designs tracking injury and performance longevity, and investigation of periodized models that strategically combine UT and BT.

## 5 Conclusion

In conclusion, this meta-analysis demonstrates that the choice between UT and BT is not a matter of overall superiority, but one of task-specific adaptation. UT is the modality of choice for enhancing unilateral power and basketball-specific cutting agility, while BT delivers comparable improvements in bilateral explosive power and linear speed. These findings provide a clear, evidence-based framework for strength and conditioning professionals to design targeted, basketball-specific training programs for male athletes.

## Data Availability

The original contributions presented in the study are included in the article/**Supplementary Material**. Further inquiries can be directed to the corresponding author.

## References

[B1] Andersen V. Fimland M. Brennset Ø. Haslestad L. R. Lundteigen M. S. Skalleberg K. . (2014). Muscle activation and strength in squat and Bulgarian squat on stable and unstable surface. Int. J. Sports Med. 35, 1196–1202. doi: 10.1055/s-0034-1382016 25254898

[B2] Appleby B. B. Cormack S. J. Newton R. U. (2019). Specificity and transfer of lower-body strength: influence of bilateral or unilateral lower-body resistance training. J. Strength Cond Res. 33, 318–326. doi: 10.1519/jsc.0000000000002923 30688873

[B3] Appleby B. B. Cormack S. J. Newton R. U. (2020). Unilateral and bilateral lower-body resistance training does not transfer equally to sprint and change of direction performance. J. Strength Cond Res. 34, 54–64. doi: 10.1519/jsc.0000000000003035 30844983

[B4] Aztarain-Cardiel K. Garatachea N. Pareja-Blanco F. (2025). Effects of bilateral and unilateral plyometric training on physical performance in male postpubertal basketball players. Int. J. Sports Physiol. Perform. doi: 10.1123/ijspp.2024-0208 39870072

[B5] Belegišanin B. Andrić N. Jezdimirović Stojanović T. Madić D. Obradović J. Radanović D. . (2025). A comparison of bilateral vs. unilateral flywheel strength training on physical performance in youth male basketball players. J. Funct. Morphol. Kinesiol 10, 81. doi: 10.3390/jfmk10010081 40137333 PMC11943462

[B6] Bobbert M. F. de Graaf W. W. Jonk J. N. Casius L. J. R. (2006). Explanation of the bilateral deficit in human vertical squat jumping. J. Appl. Physiol. 100, 493–499. doi: 10.1152/japplphysiol.00637.2005 16239616

[B7] Bullock G. S. Räisänen A. M. Martin C. Martin M. Galarneau J. M. Whittaker J. L. . (2025). Prevention strategies for lower extremity injury: a systematic review and meta-analyses for the Female, woman and/or girl Athlete Injury pRevention (FAIR) consensus. Br. J. Sports Med. 59, 1575–1586. doi: 10.1136/bjsports-2025-109910 40645751

[B8] Calle O. Mancha-Triguero D. Recio E. Ibáñez S. J. (2025). Physical fitness profiling of youth basketball players by developmental stage: A case study. J. Funct. Morphol. Kinesiol 10, 382. doi: 10.3390/jfmk10040382 41133572 PMC12551038

[B9] Cao J. C. Xun S. H. Zhang R. Li Z. Gao Y. Li A. (2024). Effects of unilateral, bilateral and combined plyometric jump training on asymmetry of muscular strength and power, and change-of-direction in youth male basketball players. J. Sports Sci. Med. 23, 754–766. doi: 10.52082/jssm.2024.754 39649573 PMC11622047

[B10] Cao S. Li Z. Wang Z. Zhang Y. Liu Y. (2025). The effects of high-intensity interval training on basketball players: a systematic review and meta-analysis. J. Sports Sci. Med. 24, 31–45. doi: 10.37766/inplasy2024.4.0058 40046212 PMC11877297

[B11] Chen J.-R. Ning Y.-L. An T.-Y. Wu C.-H. Lin Y.-C. (2025). Effects of horizontal and vertical vector resistance training on swim start performance: an eight-week intervention in division one collegiate swimmers in Taiwan. J. Funct. Morphol. Kinesiol 10, 236. doi: 10.3390/jfmk10030236 40700172 PMC12286000

[B12] Cooley C. Simonson S. R. Maddy D. A. (2024). The force-vector theory supports use of the laterally resisted split squat to enhance change of direction. J. Strength Cond Res. 38, 835–841. doi: 10.1519/jsc.0000000000004706 38662881 PMC11042517

[B13] DeForest B. A. Cantrell G. S. Schilling B. K. (2014). Muscle activity in single- vs. double-leg squats. Int. J. Exerc Sci. 7, 302–310. doi: 10.70252/mxvz7653 27182408 PMC4831851

[B14] Duan T. He Z. Dai J. Zhang Z. Zhong Y. Wang L. . (2024). Effects of unilateral and bilateral contrast training on the lower limb sports ability of college basketball players. Front. Physiol. 15, 1452751. doi: 10.3389/fphys.2024.1452751 39651433 PMC11621075

[B15] Eliassen W. Saeterbakken A. H. van den Tillaar R. (2018). Comparison of bilateral and unilateral squat exercises on barbell kinematics and muscle activation. Int. J. Sports Phys. Ther. 13, 871–881. doi: 10.26603/ijspt20180871 30276019 PMC6159498

[B16] Ferioli D. Rampinini E. Bosio A. La Torre A. Maffiuletti N. A. (2019). Peripheral muscle function during repeated changes of direction in basketball. Int. J. Sports Physiol. Perform. doi: 10.1123/ijspp.2018-0366 30427248

[B17] Fitzpatrick D. A. Cimadoro G. Cleather D. J. (2019). The magical horizontal force muscle? A preliminary study examining the “force-vector” theory. Sports 7, 30. doi: 10.3390/sports7020030 30678251 PMC6409580

[B18] Flanagan E. P. Comyns T. M. (2008). The use of contact time and the reactive strength index to optimize fast stretch-shortening cycle training. Strength Cond J. 30, 32–38. doi: 10.1519/ssc.0b013e318187e25b 38604988

[B19] Gonzalo-Skok O. Arede J. Dos' Santos T. (2025). Effects of strength training and detraining considering maturity status in youth highly trained basketball players. PloS One 20, e0317879. doi: 10.1371/journal.pone.0317879 39937825 PMC11819605

[B20] Gonzalo-Skok O. Sánchez-Sabaté J. Izquierdo-Lupón L. Silió-Román L. Pardo-Ibáñez A. (2019). Influence of force-vector and force application plyometric training in young elite basketball players. Eur. J. Sport Sci. 19, 305–314. doi: 10.1080/17461391.2018.1502357 30058461

[B21] Gonzalo-Skok O. Tous-Fajardo J. Suarez-Arrones L. Arjol-Serrano J. L. Casajús J. A. Mendez-Villanueva A. (2017). Single-leg power output and between-limbs imbalances in team-sport players: unilateral versus bilateral combined resistance training. Int. J. Sports Physiol. Perform. 12, 106–114. doi: 10.1123/ijspp.2015-0743 27140680

[B22] Hassan A. K. (2025). FITLIGHT training and its influence on visual-motor reactions and dribbling speed in female basketball players: Prospective evaluation study. JMIR Serious Games 13, e70519. doi: 10.2196/70519 40614262 PMC12252139

[B23] Hewett T. E. Myer G. D. Ford K. R. Heidt R. S. Colosimo A. J. McLean S. G. . (2005). Biomechanical measures of neuromuscular control and valgus loading of the knee predict anterior cruciate ligament injury risk in female athletes: a prospective study. Am. J. Sports Med. 33, 492–501. doi: 10.1177/0363546504269591 15722287

[B24] Jòdar-Portas A. López-Ros V. Prats-Puig A. Beltran-Garrido J. V. Madruga-Parera M. Romero-Rodríguez D. . (2023). Validity and reliability of the V-Cut dribbling test in young basketball players. Int. J. Sports Physiol. Perform. 18, 660–666. doi: 10.1123/ijspp.2022-0207 37185455

[B25] Kidgell D. J. Stokes M. A. Pearce A. J. (2011). Strength training of one limb increases corticomotor excitability projecting to the contralateral homologous limb. Mot Control 15, 247–266. doi: 10.1123/mcj.15.2.247 21628728

[B26] Koyama T. Rikukawa A. Nagano Y. Sasaki T. Isokawa M. Akiyama K. . (2022). Acceleration profile of high-intensity movements in basketball games. J. Strength Cond Res. 36, 1715–1719. doi: 10.1519/jsc.0000000000003699 32639378

[B27] Li Z. Lei S. Zhang H. Gao Y. Li A. (2025). Effects of unilateral and bilateral plyometrics training on basketball players’ leg power and change of direction ability. Sport Sci. Health. doi: 10.1007/s11332-025-01390-1 30311153

[B28] Liao K.-F. Nassis G. Bishop C. Yang W. Bian C. Feng L.-Q. . (2022). Effects of unilateral vs. bilateral resistance training interventions on measures of strength, jump, linear and change of direction speed: a systematic review and meta-analysis. Biol. Sport 39, 485–497. doi: 10.5114/biolsport.2022.107024 35959319 PMC9331349

[B29] Maestroni L. Turner A. Read P. Bettariga F. Fenu G. Rosalia A. . (2026). Interlimb asymmetry data in athletes with anterior cruciate ligament reconstruction: a comparison of different equations to interpret between-limb difference data. J. Strength Cond Res. 40, 158–166. doi: 10.1519/jsc.0000000000005280 41359911

[B30] McQuilliam S. J. Clark D. R. Erskine R. M. Brownlee T. E. (2020). Free-weight resistance training in youth athletes: A narrative review. Sports Med. 50, 1567–1580. doi: 10.1007/s40279-020-01307-7 32578028 PMC7441088

[B31] Moran J. Ramirez-Campillo R. Liew B. Chaabene H. Behm D. G. García-Hermoso A. . (2021). Effects of bilateral and unilateral resistance training on horizontally orientated movement performance: a systematic review and meta-analysis. Sports Med. 51, 225–242. doi: 10.1007/s40279-020-01367-9 33104995

[B32] Morin J.-B. Gimenez P. Edouard P. Arnal P. Jiménez-Reyes P. Samozino P. . (2015). Sprint acceleration mechanics: the major role of hamstrings in horizontal force production. Front. Physiol. 6, 404. doi: 10.3389/fphys.2015.00404 26733889 PMC4689850

[B33] MoTrPAC Study Group Lead Analysts MoTrPAC Study Group (2024). Temporal dynamics of the multi-omic response to endurance exercise training. Nature 629, 174–183. doi: 10.1038/s41586-023-06877-w 38693412 PMC11062907

[B34] Pamuk Ö. Makaracı Y. Ceylan L. Sögüt M. Kirazcı S. Clemente F. (2023). Associations between force-time related single-leg counter movement jump variables, agility, and linear sprint in competitive youth male basketball players. Children 10, 427. doi: 10.3390/children10030427 36979986 PMC10047756

[B35] Ramírez-Delacruz M. Bravo-Sánchez A. Esteban-García P. Abt G. Jiménez F. (2022). Effects of plyometric training on lower body muscle architecture, tendon structure, stiffness and physical performance: a systematic review and meta-analysis. Sports Med. - Open 8, 40. doi: 10.1186/s40798-022-00431-0 35312884 PMC8938535

[B36] Shalom A. Gottlieb R. Alcaraz P. E. González-Cutre D. Bishop C. Halfpaap J. . (2023). A narrative review of the dominant physiological energy systems in basketball and the importance of specificity and uniqueness in measuring basketball players. Appl. Sci. 13, 12849. doi: 10.3390/app132312849 30654563

[B37] Shi L. Lyons M. Duncan M. Chen S. Chen Z. Guo W. . (2022). Effects of variable resistance training within complex training on neuromuscular adaptations in collegiate basketball players. J. Hum. Kinet. 84, 174–183. doi: 10.2478/hukin-2022-0094 36457466 PMC9679182

[B38] Shin H. W. Sohn Y. H. Hallett M. (2009). Hemispheric asymmetry of surround inhibition in the human motor system. Clin. Neurophysiol. 120, 816–819. doi: 10.1016/j.clinph.2009.02.004 19299196

[B39] Spiteri T. Nimphius S. Hart N. H. Specos C. Sheppard J. M. Newton R. U. (2014). Contribution of strength characteristics to change of direction and agility performance in female basketball athletes. J. Strength Cond Res. 28, 2415–2423. doi: 10.1519/jsc.0000000000000547 24875426

[B40] Stojanović E. Scanlan A. T. Radovanović D. Jakovljević V. Živković V. Faude O. . (2025). Injury incidence rate according to mechanism, body location, and type in basketball players: A systematic review and meta-analysis. Sports Med. 55, 3093–3110. 41091317 10.1007/s40279-025-02334-y

[B41] Struzik A. Pietraszewski B. Zawadzki J. (2014). Biomechanical analysis of the jump shot in basketball. J. Hum. Kinet. 42, 73–79. doi: 10.2478/hukin-2014-0062 25414741 PMC4234772

[B42] Tao M. Nassis G. P. Song Y. Yin M. Zhu C. Lyu M. . (2025). Impact of training volume settings between unilateral training and bilateral training on athletic performance: a systematic review and meta-analysis. J. Exerc Sci. Fit 23, 291–298. doi: 10.1016/j.jesf.2025.06.003 40689312 PMC12275932

[B43] Thapa R. K. Chaware U. Sarmah B. Singh L. Singh A. (2024). The effects of single and combined jump exercises utilizing fast and slow stretch-shortening cycle on physical fitness measures in healthy adult males: a randomized controlled trial. Montenegrin J. Sports Sci. Med. 13, 25–34. doi: 10.26773/mjssm.240308

[B44] Theodorou A. S. Rizou H. P. Zacharakis E. Ktistakis I. Bekris E. Panoutsakopoulos V. . (2022). Pivot step jump: a new test for evaluating jumping ability in young basketball players. J. Funct. Morphol. Kinesiol 7, 116. doi: 10.3390/jfmk7040116 36547662 PMC9783850

[B45] Villarreal E. S.-S. Kellis E. Kraemer W. J. Izquierdo M. (2009). Determining variables of plyometric training for improving vertical jump height performance: a meta-analysis. J. Strength Cond Res. 23, 495–506. doi: 10.1519/jsc.0b013e318196b7c6 19197203

[B46] Wang P. Liu Y. Chen C. (2024). Effects of neuromuscular training on dynamic balance ability in athletes: a systematic review and meta-analysis. Heliyon 10, e36434. doi: 10.1016/j.heliyon.2024.e35823 39220942 PMC11365420

[B47] Young W. James R. Montgomery I. (2002). Is muscle power related to running speed with changes of direction? J. Sports Med. Phys. Fitness 42, 282–288. 12094116

[B48] Zemkova E. Hamar D. (2010). The effect of 6-week combined agility-balance training on neuromuscular performance in basketball players. J. Sports Med. Phys. Fitness 50, 262–267. 20842085

[B49] Zhang W. Chen X. Xu K. Gao Y. Wang S. Wang Z. . (2023). Effect of unilateral training and bilateral training on physical performance: a meta-analysis. Front. Physiol. 14, 1128250. doi: 10.3389/fphys.2023.1128250 37123275 PMC10133687

[B50] Zhang W. Chen X. Xu K. Gao Y. Wang S. Wang Z. . (2024). The potential of a targeted unilateral compound training program to reduce lower limb strength asymmetry and increase performance: a proof-of-concept in basketball. Front. Physiol. 15, 1361719. doi: 10.3389/fphys.2024.1361719 38989050 PMC11234801

[B51] Zhang Z. Jiang M. Jing Y. Li M. Li Y. Yang X. (2024). Associations between sprint mechanical properties and change of direction ability and asymmetries in COD speed performance in basketball and volleyball players. Life. (Basel) 14, 1434. doi: 10.3390/life14111434 39598231 PMC11595913

[B52] Zhang Z. Qu W. Peng W. Wang G. Wang L. Wang Y. . (2025). Effect of unilateral and bilateral plyometric training on jumping, sprinting, and change of direction abilities: a meta-analysis. BMC Sports Sci. Med. Rehabil. 17, 97. doi: 10.1186/s13102-025-01113-6 40281589 PMC12032719

[B53] Zhao Y. X. Sun M. M. Wang X. S. Li Z. Gao Y. Li A. (2024). Unilateral plyometric jump training shows significantly more effective than bilateral training in improving both time to stabilization and peak landing force in single-leg lend and hold test: a randomized multi-arm study conducted among young male basketball players. J. Sports Sci. Med. 23, 647–658. doi: 10.52082/jssm.2024.647 39228781 PMC11366841

[B54] Zhiguang W. (2022). Improvement effect of different types of basketball specific motion training on athletes' explosive strength and sensitivity study. Rev. Multidiscip. Cienc. Deporte 22, 513–540.

[B55] Zhou J. Y. Wang X. Hao L. Li Z. (2024). Meta-analysis of the effect of plyometric training on the athletic performance of youth basketball players. Front. Physiol. 15, 1427291. doi: 10.3389/fphys.2024.1427291 39376898 PMC11457583

