# Supplementary material for: Unilateral versus bilateral resistance training for explosive jump performance, linear sprint speed, and change-of-direction ability in male basketball players: a systematic review and meta-analysis
Source: Front Physiol. 2026 Jun 15;17:1798477. doi: 10.3389/fphys.2026.1798477 (PMC13310738; doi:10.3389/fphys.2026.1798477)
Supplement: Supplementary file 1 [file DataSheet1.pdf]

Fig 1 The Preferred Reporting Items for Systematic Reviews and Meta-Analyses flow diagram.

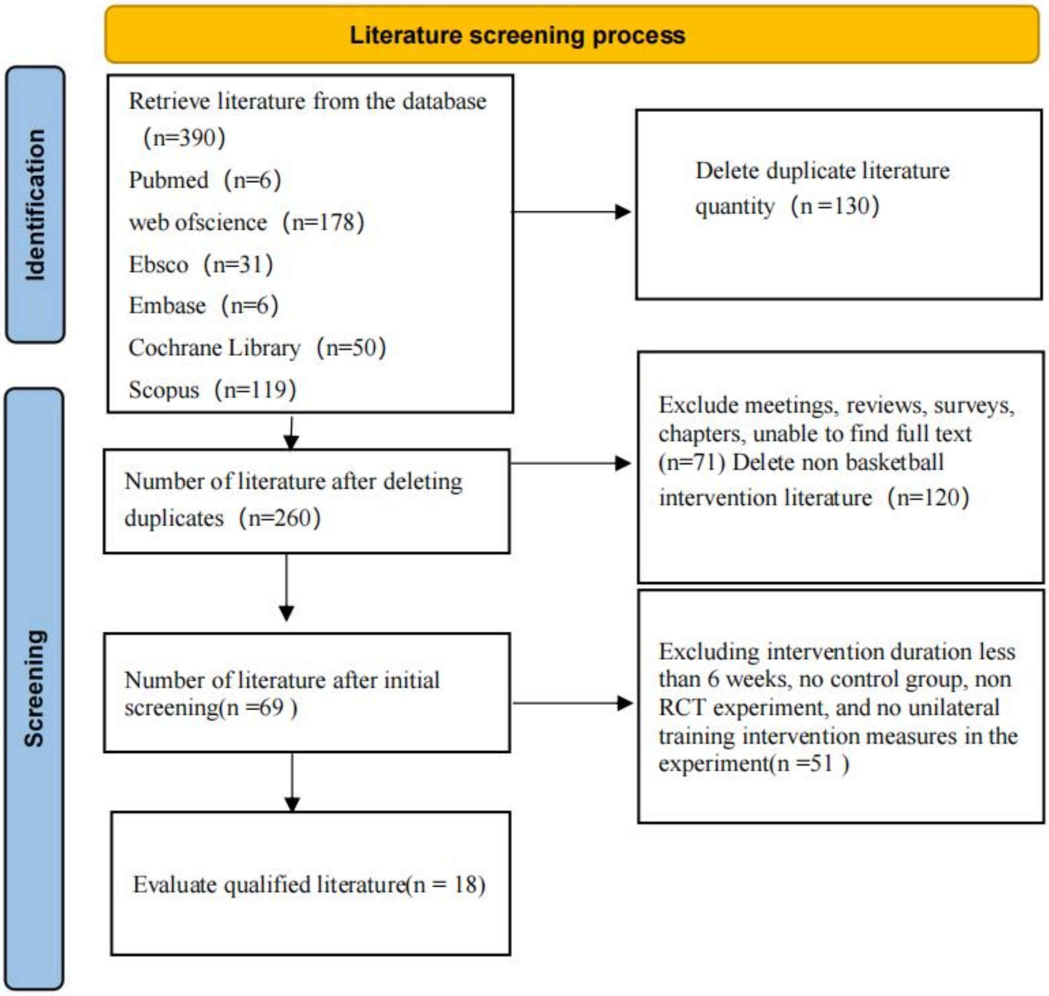

Fig 2. Risk of bias summary: A review of the authors' judgments about the risk of bias of each item in each included study.

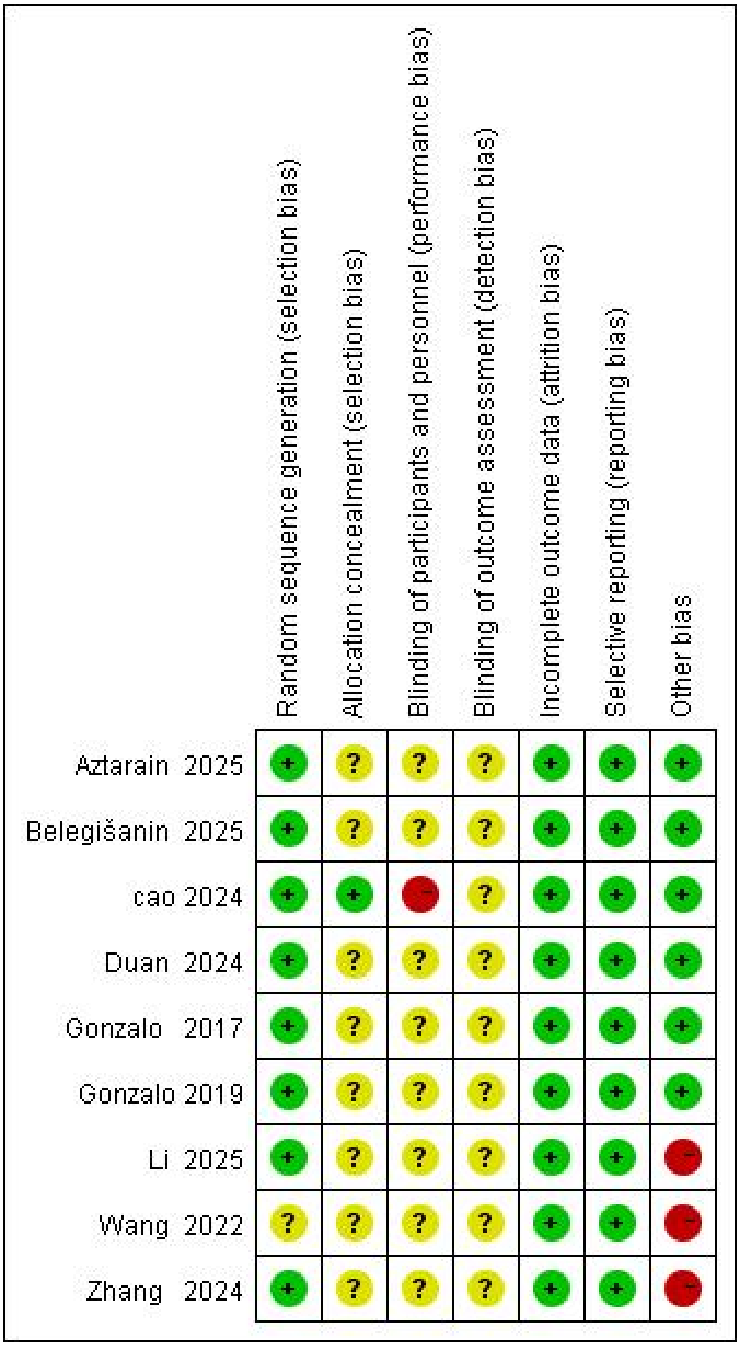

Table 1 Basic characteristics of the control group included in the study.

Study	Age(years)	Weight(kg)	Height(cm)	Sample size		Exercise<br>Content Sets/Repetitions	Frequency andWeek	Test used
Gonzalo 2019	13.3 ± 06	59.1 ± 12.8	172.8 ± 7.9	9	9	Drop Jump 20 cm 3 × 5<br>SJ with arms swing 2 × 5<br>CMJ with arms swing 2 × 5<br>Tuck Jump 5 × 2<br>Hurdle jumps 3 × 5	2/6week	CMJ, 5 meter sprint, Single-leg CMJ, V-shaped directional change
Duan 2024	20.80 ± 1.14	76.20 ± 8.70	183.00 ± 5.86	10	10	Barbell Back Squat 6x5<br>Bilateral Vertical Jump 6x10	2/8week	CMJ, 20 meter sprint, 505 Agility Test
Aztarain 2025	14.2 (1.1)	182.3 (9.1)	68.3 (11.6)	15	15	Vertical acyclic (bilateral)<br>Horizontal acyclic (bilateral)<br>Vertical cyclic (bilateral)<br>Horizontal cyclic (bilateral)<br>Total volume (contacts/session)	2/6week	CMJ, Single-leg CMJ, V-shaped directional change, 20 meter sprint
Zhang 2024	20.9 ± 0.9	69.1 ± 6.7	178.7 ± 5.8	15	15	Routine Training Plan for Both Lower Limbs	3/10week	CMJ
Cao 2024	16.3 ± 0.8	68.9 ± 6.7	180.5 ± 5.0	16	16	Bilateral reactive pogo jumps (2x10);<br>bilateral countermovement jumps (2x10);<br>bilateral drop jumps at 10 cm (2x10)<br>Bilateral horizontal jumps (2x10);<br>bilateral three consecutive horizon jumps (2x10)	2/8week	Single-leg CMJ
Gonzalo 2017	16.7 ± 1.7	74.9 ± 9.6	188.9 ± 7.5	9	9	Bilateral 90° Deep Squat 2x5<br>Bilateral Vertical Jump 2x5<br>Jump in place with both sides 2x5	2/6week	CMJ, 5 meter sprint, V-shaped directional change
Wang 2022	20.4±1.35	80.71±5.02	186.1±4.06	10	10	Vertical, horizontal, and forward leg jumps 2x4	2/8week	CMJ, Single-leg CMJ, V-shaped directional change, RSI
Belegišanin	15.2 ± 0.4	69 ± 7	183 ± 4	11	11	Half Squat (Flywheel Device) 6x6	2/6week	CMJ, Single-leg CMJ, RSI, 20

Study	Age(years)	Weight(kg)	Height(cm)	Sample size		Exercise Content	Exercise Sets/Repetitions	Frequency andWeek	Test used
2025									meter sprint, 5 meter sprint, 505 Agility Test
Li 2025	20.4 ±1.4	80.7 ±5.0	186.1 ±4.1	10	10	B vertical jump and freezing 2x8<br>B standing long jump and freezing 2x8<br>B consecutive vertical jump 2x8		2/8week	CMJ, Single-leg CMJ, V-shaped directional change, RSI, 505 Agility Test

Table 2 Basic characteristics of the experimental group included in the study

Study	Age(years)	Weight(kg)	Height(cm)	Sample size		Exercise Content	Exercise Sets/Repetitions	Frequency andWeek	Test used
Gonzalo 2019	13.3 ± 06	59.6 ± 11.7	171.7 ± 7.2cm	9	9	Drop Jump 10 cm3 × 5<br>SLJ2 × 5<br>SLJ without CMJ2 × 5<br>Unilateral jumps 5 × 2<br>Triple jumps<br>3 × 5		2/6week	CMJ, 5 meter sprint, Single-leg CMJ, V-shaped directional change
Duan 2024	19.90 ± 1.45	77.90 ± 5.90	183.20 ± 6.82	10	10	Bulgarian Split Squat 5x6<br>Reverse Lunge Jump 10x5		2/8week	CMJ, 20 meter sprint, 505 Agility Test
Zhang 2024	20.9 ± 1.1	73.5 ± 5.3	182.1 ± 4.1	15	15	Bulgarian split squat 3x6<br>Box step-up 3x12<br>Single-leg calf raise 1x12<br>Plyometric jump 1x12<br>Single-leg jump with rear leg lift 1x12<br>Single-leg lateral jump 1x12<br>Single-leg consecutive jumps 1x12		3/10week	CMJ
Gonzalo	16. 8 ± 1.7	76.9 ± 8.6	190.4 ± 6.9	9	9	Single-Leg 90° Deep Squat 3x5		2/6week	CMJ, 5 meter sprint, V-shaped

Study	Age(years)	Weight(kg)	Height(cm)	Sample size		Exercise Content Sets/Repetitions	Frequency andWeek	Test used
2017						Single-leg vertical jump 2x5<br>Single-Side Jump from the Spot 2x5		directional change
Aztarain 2025	14.1 (1.3)	69.1±9.3	181.1 ±8.0	15	15	Vertical acyclic<br>Horizontal acyclic<br>Vertical cyclic (unilateral)<br>Horizontal cyclic (unilateral)	2/6week	CMJ, Single-leg CMJ, V-shaped directional change, 20 meter sprint
Belegišanin 2025	15.5 ± 0. 5	71 ± 12	186 ± 6	11	11	Split Squat (Flywheel Device)6x6	2/6week	CMJ, Single-leg CMJ, RSI, 20 meter sprint, 5 meter sprint, 505 Agility Test
Cao 2024	15.9 ± 0.9	62.4 ± 5.2	175.5 ± 6.9	16	16	Single-Sided Lateral Jump 2x5<br>Single-Sided Triple Jump 2x5<br>Single-leg reactive jump 2x5<br>Single-Leg Squat Jump 2x10	2/8week	Single-leg CMJ
Wang 2022	20.6±1.51	79.95±6.56	185.6±3.63	10	10	Single-leg landing jump 2x10<br>Single-leg jumps: vertical, horizontal, and forward	2/8week	CMJ, Single-leg CMJ, V-shaped directional change, RSI
Li 2025	20.6 ± 1.5	79.9 ±6.6	185.6 ±3.6	10	10	U vertical jump and freezing 2x8<br>U standing long jump and freezing 2x8<br>U consecutive vertical jump 2x8	2/8week	CMJ, Single-leg CMJ, V-shaped directional change, RSI, 505 Agility Test

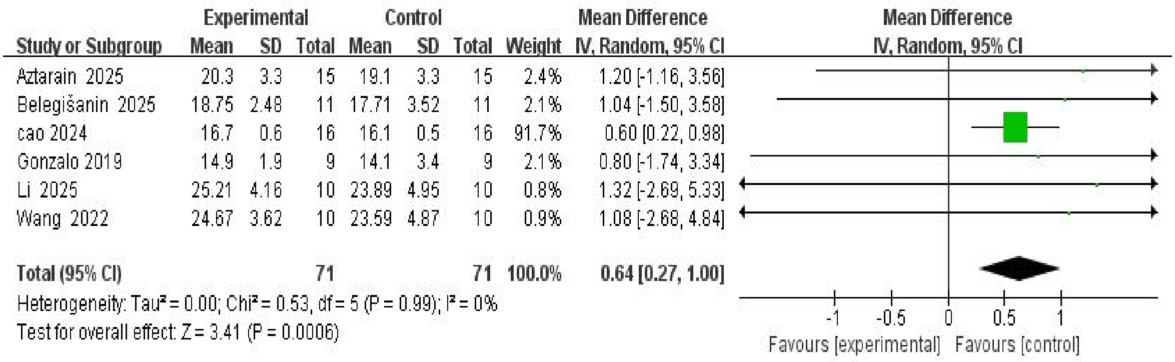

Fig 3 Forest plot of single-leg and double-leg performance in single-leg CMJ

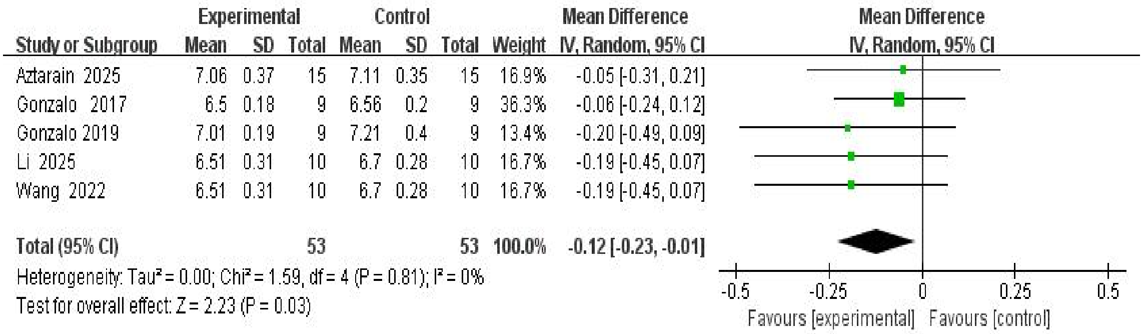

Fig 4 Forest plot of single-leg and double-leg performance in V-shaped directional change

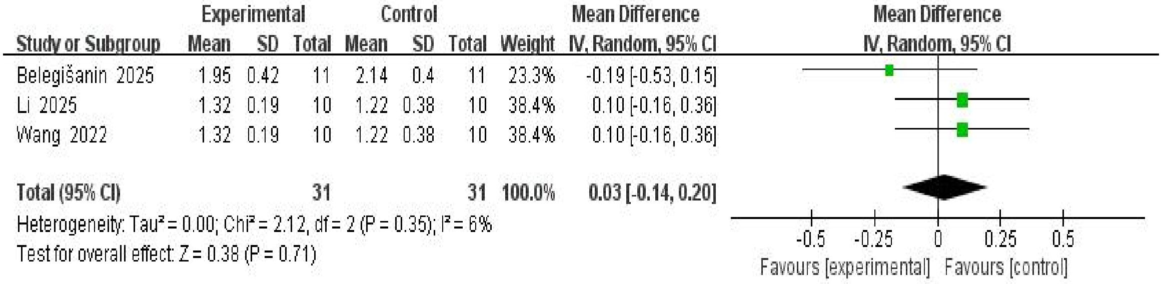

Fig 5 Forest plot of single-leg and double-leg performance in RSI

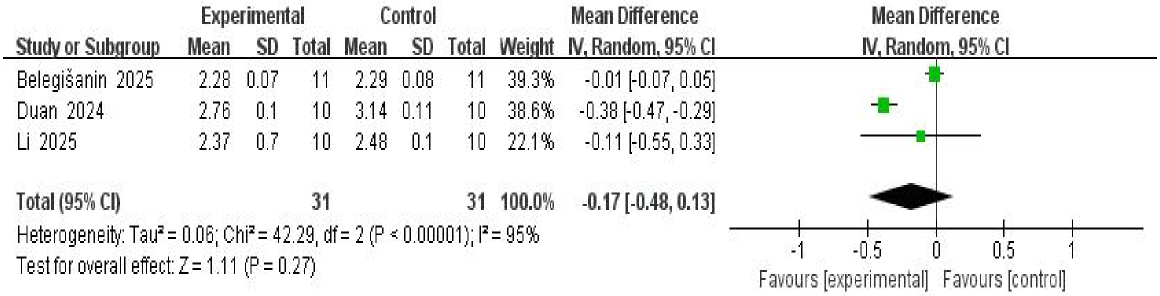

Fig 6 Forest plot of single-leg and double-leg performance in 505 Agility

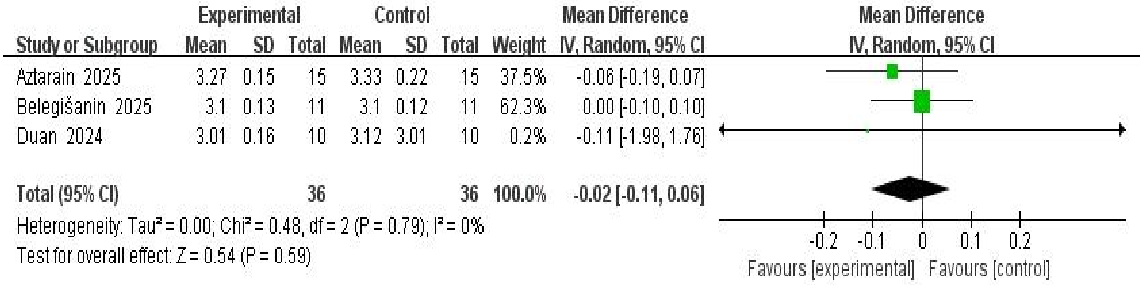

Fig 7 Forest plot of single-leg and double-leg performance in 20 meter sprint

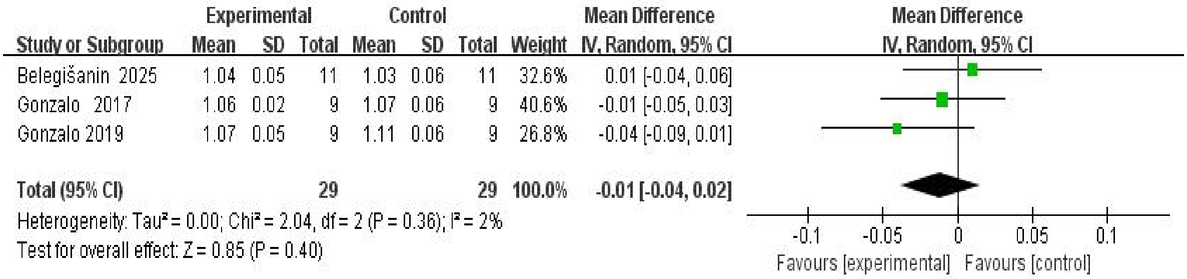

Fig 8 Forest plot of single-leg and double-leg performance in 5 meter sprint

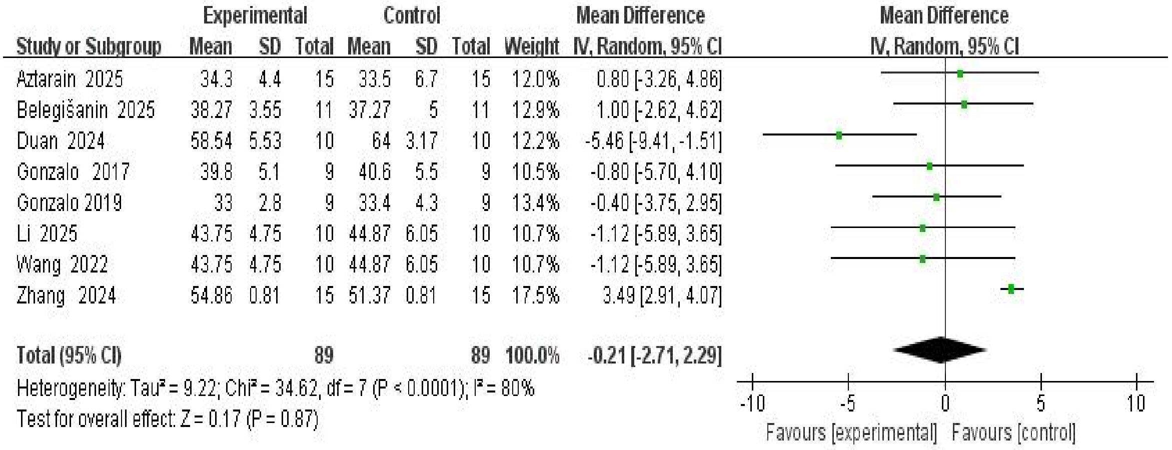

Fig 9 Forest plot of single-leg and double-leg performance in CMJ

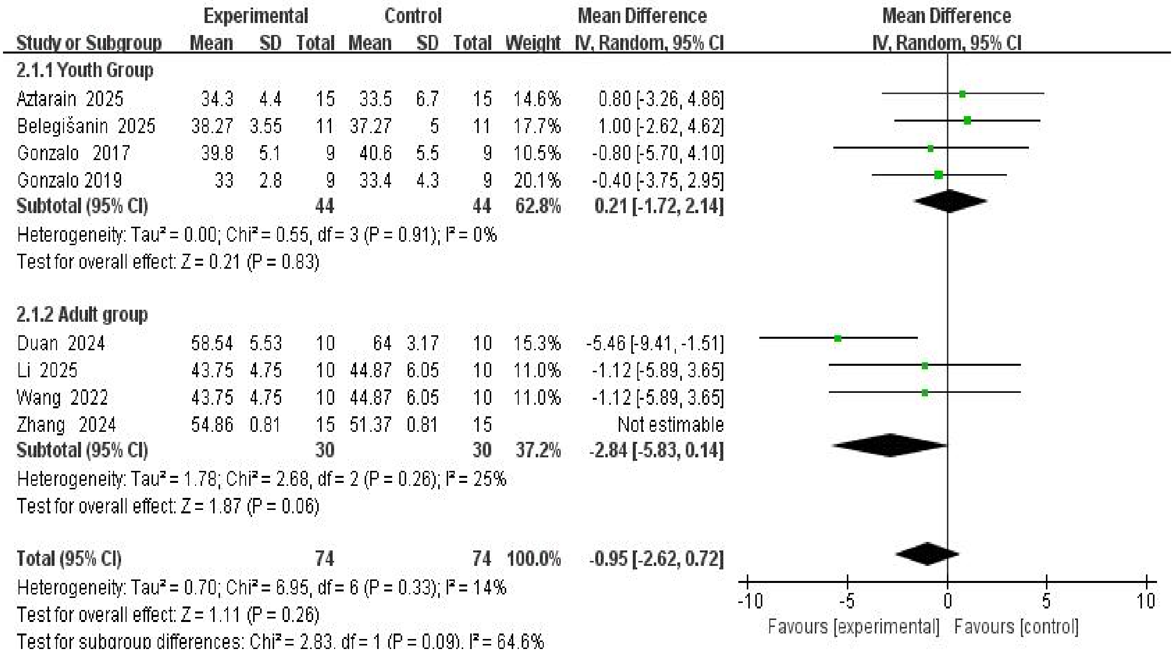

Fig 10 Subgroup analysis in CMJ test

Table 1. Summary of findings (GRADE) for meta-analysis outcomes

Outcome	Studies (k)	Participants (n)	Effect estimate (SMD, 95% CI)	Certainty of evidence (GRADE)	Comments / reasons for downgrading
Single-leg CMJ	6	142	SMD = 0.64 (95% CI 0.27 to 1.00)	⊕⊕⊕◯ MODERATE (1)	due to risk of bias
V-shaped directional change	5	106	SMD = -0.12 (95% CI -0.23 to -0.01)	⊕⊕⊕◯ MODERATE (1)	due to risk of bias
RSI	3	62	SMD = 0.03 (95% CI -0.14 to 0.20)	⊕⊕◯◯ LOW (1,2)	due to risk of bias, imprecision
505 Agility Test	3	62	SMD = -0.17 (95% CI -0.48 to 0.13)	⊕◯◯◯ VERY LOW (1,2,3)	due to risk of bias, inconsistency, imprecision
20 meter sprint	3	72	SMD = -0.02 (95% CI -0.11 to 0.06)	⊕⊕◯◯ LOW (1,2)	due to risk of bias, imprecision
5 meter sprint	3	58	SMD = -0.01 (95% CI -0.04 to 0.02)	⊕⊕◯◯ LOW (1,2)	due to risk of bias, imprecision
CMJ	8	178	SMD = -0.21 (95% CI -2.71 to 2.29)	⊕◯◯◯ VERY LOW (1,2,3)	due to risk of bias, inconsistency, imprecision
