# Supplementary material for: Unilateral versus bilateral resistance training for explosive jump performance, linear sprint speed, and change-of-direction ability in male basketball players: a systematic review and meta-analysis
Source: Front Physiol. 2026 Jun 15;17:1798477. doi: 10.3389/fphys.2026.1798477 (PMC13310738; doi:10.3389/fphys.2026.1798477)
Supplement: Supplementary file 2 [file DataSheet2.pdf]

Boolean search strings

All databases were searched from inception to 5 April 2026.

**Embase (Elsevier)**	**('unilateral exercises':ti,ab,kw OR 'unilateral training':ti,ab,kw OR 'single-limb training':ti,ab,kw OR 'asymmetrical loading':ti,ab,kw OR 'unilateral resistance training':ti,ab,kw OR 'bilateral training':ti,ab,kw) AND ('basketball'/exp OR basketball:ti,ab,kw)**
**Cochrane Library (Wiley)**	**(("unilateral exercises" OR "unilateral training" OR "single-limb training" OR "asymmetrical loading" OR "unilateral resistance training" OR "bilateral training"):ti,ab,kw) AND (MeSH descriptor: [Basketball] explode all trees OR basketball:ti,ab,kw)**
**Scopus(Elsevier)**	**TITLE-ABS-KEY(("unilateral exercises" OR "unilateral training" OR "single-limb training" OR "asymmetrical loading" OR "unilateral resistance training" OR "bilateral training") AND basketball)**
**CINAHL via EBSCOhost**	**AB (("unilateral exercises" OR "unilateral training" OR "single-limb training" OR "asymmetrical loading" OR "unilateral resistance training" OR "bilateral training") AND basketball)**
**Web of science Core Collection (Clarivate)**	**TS=(("unilateral exercises" OR "unilateral training" OR "single-limb training" OR "asymmetrical loading" OR "unilateral resistance training" OR "bilateral training") AND basketball)**
**PubMed (NLM)**	**(("unilateral exercises"[Title/Abstract] OR "unilateral training"[Title/Abstract] OR "single-limb training"[Title/Abstract] OR "asymmetrical loading"[Title/Abstract] OR "unilateral resistance training"[Title/Abstract] OR "bilateral training"[Title/Abstract]) AND "basketball"[Title/Abstract])**
